# Distinct circular RNA signatures distinguish STEMI from NSTEMI in acute myocardial infarction

**DOI:** 10.3389/fcvm.2026.1913930

**Published:** 2026-09-25

**Authors:** Mohammed Almansori, Rudaynah A. Alali, Naif Khalid Alqannas, Alawi H. Habara, Ali Alsaeed, Chittibabu Vatte, Cyril Cyrus, Abrar J. Alwaheed, Hassan Albisher, Almujtaba Alshangity, Pauline Dergham, Abir Hamad Alsaid, Moyad M. Almuslim, Morten T. Venø, Brendan J. Keating, Amein K. Al-Ali

**Affiliations:** 1 Department of Internal Medicine, Cardiology Section, King Fahd Hospital of the University, Alkhobar, Imam. Abdulrahman bin Faisal, Dammam, Saudi Arabia; 2 Department of Adult Cardiology, Saud Al Babtain Cardiac Center, Dammam, Saudi Arabia; 3 Department of Biochemistry, College of Medicine, Imam Abdulrahman bin Faisal University, Dammam, Saudi Arabia; 4 Department of Cardiology, Dammam Medical Complex, Dammam, Saudi Arabia; 5 Department of Internal Medicine, King Fahd Hospital of the University, Alkhobar, Imam Abdulrahman bin Faisal, Dammam, Saudi Arabia; 6 Department of Surgery, King Fahd Hospital of the University, Alkhobar, Imam Abdulrahman bin Faisal, Dammam, Saudi Arabia; 7 Department of Internal Medicine, College of Medicine, Northern Border University, Arar, Saudi Arabia; 8 College of Medicine, Imam Abdulrahman bin Faisal University, Dammam, Saudi Arabia; 9 College of Pharmacy, Imam Abdulrahman bin Faisal, Dammam, Saudi Arabia; 10 Department of Cardiology, Omics ApS, Åbogade, Aarhus N, Denmark; 11 Department of Surgery, NYU Grossman School of Medicine, New York, NY, United States

**Keywords:** biomarker, circRNA, circular RNA, host-gene expression, myocardial infarction, NSTEMI, RNA sequencing, Saudi arabia

## Abstract

**Introduction:**

Heart attacks come in different forms, with ST-elevation myocardial infarction (STEMI) and non-ST-elevation myocardial infarction (NSTEMI) being two important but distinct types. While doctors can tell them apart in the clinic, it’s still unclear exactly how the molecules circulating in the blood differ between the two. Recently, scientists have been looking at circular RNAs (circRNAs), a type of stable non-coding RNA easily found in blood plasma, to see if they can offer new clues beyond traditional heart attack markers. This study set out to find out if the patterns of circRNAs in the blood could reliably tell STEMI and NSTEMI apart, and possibly uncover new, more specific biomarkers for each type.

**Methods:**

This exploratory observational study included 59 patients with STEMI, 78 with NSTEMI, and 109 hospital-based controls recruited from two hospitals in Eastern Saudi Arabia. Blood samples were collected in EDTA tubes within two hours after the diagnosis of acute myocardial infarction. Researchers then used advanced sequencing techniques to look for circRNAs in these blood samples. They compared the circRNA patterns between STEMI, NSTEMI, and control groups, making sure to account for differences in age and other factors. The team also checked how the circRNAs compared with the usual gene activity and tested how well these patterns could tell the two heart attack types apart using a standard accuracy metric.

**Results:**

A total of 297 circRNAs were differentially expressed between STEMI and NSTEMI at a false-discovery rate below 0.05. Several circRNAs, including hsa-PTPN12_000054, hsa-UBAC2_000001, hsa-STK39_000074, hsa-ERP44_000019, and hsa-LINC00299_000001, were enriched in STEMI cases. Meanwhile, circRNAs including hsa-CHD2_000062, hsa-PCCA_000138, hsa-RBM33_000015, and hsa-NPM1_000005 were more abundant in NSTEMI. An exploratory three-circRNA logistic model produced an apparent AUC of 0.866 and a five-fold cross-validated AUC of 0.813. Interestingly, these circRNA changes didn’t always match up with changes in the genes they came from, suggesting that circRNAs might be regulated in their own unique way.

**Conclusion:**

Plasma circRNA profiles differed between STEMI and NSTEMI in this exploratory discovery cohort. The identified candidates provide a basis for technical confirmation and external validation, but they should not currently be considered clinically validated diagnostic biomarkers.

## 1 Introduction

A myocardial infarction (MI), or heart attack, remains one of the major health challenges worldwide, and, alarmingly, recent studies suggest it is increasingly affecting younger adults (1, 2). MI occurs when blood flow in the coronary arteries is suddenly reduced or blocked, resulting in decreased oxygen supply to the heart muscle, impaired function, and increased risk of mortality (3). Most often, this is triggered by rupture of an atherosclerotic plaque, which leads to sudden arterial blockage and heart muscle ischemia (4). It's important to remember that atherosclerosis is not merely an acute trigger but a chronic inflammatory process that develops over many years due to injury to the artery wall, lipid buildup, and ongoing immune activation (1).

In clinical practice, MI is most commonly categorized as ST-elevation (STEMI) or non-ST-elevation (NSTEMI) myocardial infarction. Although these subtypes are sometimes lumped together, they actually differ in electrocardiographic changes, arterial involvement, the extent of heart tissue affected, and the urgency of intervention. These differences are not only diagnostic but also biologically meaningful, influencing both immediate management and long-term outcomes for patients.

What's sometimes overlooked is that the signals we detect in the blood after MI may reflect not only the damage to heart muscle but also the underlying inflammatory and unstable arterial plaque that set the event in motion. This overlap is crucial for interpreting new molecular markers and may help explain why patients with similar clinical symptoms can have different trajectories.

In daily clinical settings, the diagnosis of MI relies on a combination of symptoms, ECG changes, imaging, and cardiac biomarkers such as troponins. While troponins are invaluable for confirming myocardial injury, they cannot fully explain the upstream mechanisms responsible for the event. Unstable plaques, thrombosis, inflammation, and cellular stress all contribute, yet we are still unraveling exactly how these pieces fit together. Motivated by this gap, researchers, including our group, have increasingly focused on gene expression signatures in blood as a potential window into the molecular complexity of acute MI. Such approaches could reveal novel markers that complement conventional diagnostics and provide new insights into disease mechanisms.

Recently, circular RNAs (circRNAs) have emerged as particularly intriguing candidates in this context. Unlike linear RNAs, circRNAs form closed-loop structures that confer remarkable stability in biofluids (3–6). Their expression profiles vary by tissue and disease state, making them attractive as potential biomarkers. Far from being merely a by-product, circRNAs participate in gene regulation, interacting with other RNAs and proteins, and in some cases even encoding small peptides (4, 5, 7). CircRNAs have already been linked to a range of key processes relevant to MI, including cell survival, inflammation, blood vessel adaptation, and tissue repair (8–10). Several studies suggest that circRNA levels in blood change after MI, potentially helping clinicians to distinguish between patients at higher risk of adverse outcomes (11, 12). However, this field is still in its early days, and many questions remain about which circRNA changes are clinically meaningful, or whether there are distinct patterns between STEMI and NSTEMI.

Our previous work provided a foundation for the present study by profiling plasma circRNA landscapes in acute MI relative to healthy controls (13). We observed broad remodeling of circulating circRNAs, including several with promising biomarker potential. Functional analyses suggested that these changes were linked to inflammatory pathways, platelet activation, and other biological processes relevant to MI (13). Yet, prior studies typically considered MI as a single entity, without differentiating STEMI from NSTEMI. This distinction is important. By directly comparing circRNA profiles between STEMI and NSTEMI, we may uncover subtype-specific signatures that reflect pathophysiological differences, such as infarct severity, coronary occlusion, or unique regulatory responses. Additionally, we sought to determine whether circRNA changes simply mirror alterations in corresponding host-gene expression, or if they provide independent information as a post-transcriptional regulatory layer (4, 5). It's important to clarify that STEMI and NSTEMI are diagnosed and managed using symptoms, repeated ECGs, cardiac troponins, and coronary assessments. Circulating circRNAs are not intended to replace these established diagnostic methods. Instead, their potential is as a complementary tool, they might help uncover molecular differences, refine prognostic or treatment subgroups, or enhance multimarker models in cases where standard findings are unclear. To show that circRNAs can truly add value, future prospective studies are needed to test whether they can improve diagnosis, risk stratification, or clinical decision-making beyond what is possible with traditional variables.

Therefore, the present work moves beyond broad case-control comparisons to focus on the molecular differences between STEMI and NSTEMI. Therefore, we aimed to identify circRNAs associated with STEMI and NSTEMI, examine their relationship with host-gene expression, evaluate their exploratory subtype-discrimination performance, and place the findings in biological context through functional enrichment analysis. The study was designed as an exploratory discovery analysis rather than as the development or validation of a clinical diagnostic test. By integrating differential expression, host-gene comparison, ROC curve analysis, and functional annotation, this study seeks to clarify the additional value circRNAs may offer for understanding heterogeneity within acute MI and for identifying candidate biomarkers that complement existing clinical approaches (14).

## 2 Materials and methods

### 2.1 Study design and participants

We conducted a prospective, observational, case–control study, enrolling adults with acute myocardial infarction (MI) and hospital- based control participants. Participants were recruited from King Fahd Hospital of the University (Al-Khobar) and Dammam Medical Complex (Dammam) in Saudi Arabia's Eastern Province. MI cases were diagnosed strictly according to ACC/AHA recommendations and the Fourth Universal Definition of Myocardial Infarction, requiring a significant change in cardiac troponin (above the 99th percentile), with supporting clinical evidence such as symptoms, ECG findings, or imaging (15). This design was intended to capture the spectrum of MI typically seen in everyday practice in our region. Control subjects were identified from the same hospitals and catchment areas as cases, frequency-matched for sex and major cardiovascular risk factors, but with no prior history of MI. We encountered a practical challenge: for many older potential controls, incomplete longitudinal records made it difficult to rule out prior MI. To address this, we prioritized recruiting younger controls, accepting the resulting age imbalance as a trade-off to reduce misclassification. All participants gave written informed consent before enrollment. A consultant cardiologist reviewed the clinical data for all participants to ensure consistent application of inclusion and exclusion criteria across both sites. If any case was ambiguous, it was referred to a panel of consultants for adjudication, with extra clinical data requested when needed. Eligible participants were adults (aged 21–88 years) with a clinical diagnosis of MI, documented coronary artery disease, or a history of coronary revascularization. We excluded individuals with COPD, right-sided heart failure, chronic kidney disease requiring ongoing care, malignancy (current or prior), chest pain without objective ischemia, or first-degree relatives of those already enrolled. Blood samples were collected in EDTA tubes within two hours after the diagnosis of acute myocardial infarction. Following clinical eligibility assessment and sequencing quality control, 246 participants were included in the final analysis, comprising 59 patients with STEMI, 78 with NSTEMI, and 109 controls. Three additional participants from the initial cohort of 249 were excluded because they were diagnosed with unstable angina and therefore did not meet the prespecified myocardial infarction phenotype criteria. As this was an exploratory study based on an available discovery cohort, no prospective sample-size calculation was performed. The study was designed to identify candidate circRNA signatures for subsequent validation rather than to provide definitive estimates of diagnostic accuracy.

Blood samples were collected in EDTA tubes within two hours of the index MI event. Plasma was separated within an hour of collection and stored at −80 °C until RNA extraction and sequencing. The study protocol was approved by the Institutional Review Board of Imam Abdulrahman bin Faisal University (IRB# 2019-01-104) and the Ministry of Health, Dammam (IRB# 2023-02; March 29, 2023). All procedures followed the Declaration of Helsinki.

### 2.2 Plasma RNA isolation and library preparation

We isolated total RNA from 500 μL of plasma per sample using the miRNeasy Serum/Plasma Advanced Kit (Qiagen) and removed residual DNA with the TURBO DNA-free™ Kit (Thermo Fisher). Ribosomal RNA was depleted, and stranded RNA-seq libraries were prepared with the SMARTer Stranded Total RNA-Seq Kit v3 Pico Input Mammalian (Takara), which incorporates unique molecular indices (UMIs) for PCR duplicate removal. Library quality was assessed with an Agilent Bioanalyzer. *Libraries were pooled at* equimolar concentrations and sequenced on an Illumina NextSeq 2000 platform using a 150 bp paired-end sequencing configration.

### 2.3 Preprocessing, alignment, UMI deduplication, and quantification

Adapters and low-quality bases with Phred scores below 20 were removed using Trim Galore. Sequencing quality was evaluated using FastQC, MultiQC and Picard, and summarized across samples using MultiQC. The reviewed quality metrics included read depth, per-base sequence quality, percentage of bases with Phred scores of at least 30, adapter content, sequence duplication, library complexity, and genome-mapping rate. Samples were excluded according to prespecified criteria of having below 300 unique circRNAs detected. Reads were aligned to the hg19/GRCh37 reference genome using STAR, and PCR duplicates were removed using UMI-tools. Gene-level counts were generated using featureCounts with GENCODE version 37 annotations. CircRNAs were retained for downstream analysis when supported by at least 2 back-splice-junction reads in at least 33% of samples. Back-splice junctions were identified using CIRI2 (v2.0.6) as bundled in CIRIquant (v1.1.3), with parameters specifying the number of threads (-t), input FASTQ files (−1, −2), output directory (-o), log file (–log), and a YAML configuration providing CIRI2 access to the bwa2 and hisat2 indexes, genome FASTA, and gene annotation GTF. For internal validation, DCC (v0.5.0; https://github.com/dieterich-lab/DCC) as implemented in circtools (v1.3.1) was run for paired-end data according to the DCC documentation; circRNA candidates were not required to be detected by both algorithms, since circRNA expression used for analysis was derived from CIRI2.

To support the reliability of circRNA detection, we ran several in silico quality checks: (1) comparing back-splice junction support to linear splice support within the same genomic region, (2) assessing the correlation between circRNA and host gene mRNA expression, and (3) comparing group-wise changes in circRNA and mRNA from the same host gene. These steps helped us focus on circRNAs with strong, specific signals rather than those simply tracking host gene abundance.

### 2.4 Differential expression and statistical modeling

Differential-expression analysis was performed using DESeq2 in R with the following design formula:

design=∼Sex+Smoking+Batch+Age+Percent_mRNA_mapping+Group

This model was used to account for relevant demographic and technical differences among samples. The percentage of reads mapping to gene exons was included as a sample-level sequencing quality covariate. Gene-level linear RNA counts and circRNA back-splice-junction counts were analyzed separately using DESeq2 with the same design formula. CircRNA host-gene interpretation was based on the genomic mapping of each circRNA to its corresponding host gene. *P* values were adjusted for multiple comparisons using the Benjamini–Hochberg procedure, and an adjusted *P* value, or false discovery rate, below 0.05 was considered statistically significant unless otherwise specified.

Additional clinical variables considered as potential confounders included diabetes mellitus, hypertension, dyslipidaemia, renal function, medication exposure, time from symptom onset to blood sampling, markers of infarct size, and reperfusion treatment. However, these variables were either unavailable or had substantial missingness and therefore could not be included reliably in the multivariable differential-expression model. To preserve model stability in this discovery cohort, the final model was limited to age, sex, smoking status, sequencing batch, percentage of mRNA mapping, and clinical group. Consequently, residual confounding by unmeasured or incompletely recorded clinical variables cannot be excluded.

### 2.5 Biomarker performance

Receiver operating characteristic curves and corresponding areas under the curve were calculated using the pROC package in R. Single-circRNA ROC analyses were performed using normalized circRNA expression values as continuous predictors of STEMI versus NSTEMI status. Candidate circRNAs were shortlisted based on a combination of effect size, adjusted *P* value, consistency of expression across samples, annotation in circRNA databases, and single-marker ROC performance.

For the exploratory panel, expression values normalized using DESeq2 in R followed by variance stabilizing transformation (VST) for hsa-PTPN12_000054, hsa-LINC00299_000001, and hsa-PPP3CC_000001 were entered as continuous predictors in a binary logistic regression model with STEMI coded as [1/0]. STEMI was treated as the positive outcome and NSTEMI as the reference outcome. Predicted probabilities from the fitted model were used to construct the combined receiver operating characteristic curve. The apparent AUC was calculated by fitting and evaluating the model in the complete discovery dataset.

Internal performance was assessed using five-fold cross-validation. During each iteration, the model was fitted to four folds and evaluated using the held-out fold. Predictions for held-out participants were pooled across the five folds and used to calculate the cross-validated AUC. Candidate selection was performed using the complete discovery dataset before cross-validation; therefore, the cross-validated AUC may remain subject to optimism and was interpreted as an exploratory internal performance estimate rather than an unbiased estimate of external diagnostic performance.

### 2.6 Gene ontology and disease-association enrichment analyses

Host genes corresponding to significantly dysregulated circRNAs were evaluated for Gene Ontology enrichment using the R package clusterProfiler version 4.8.3, with all expressed genes from the RNA-seq data as background. The Biological Process, Molecular Function, and Cellular Component domains were analyzed separately using an over-representation framework. *P* values were adjusted for multiple comparisons using the Benjamini–Hochberg procedure. Terms with an adjusted *P* value below 0.05 were considered statistically significant. Enrichment plots display the most statistically significant terms ranked according to their adjusted *P* values.

Disease-association enrichment was performed using DisGeNET annotations applied to the host genes of significantly dysregulated circRNAs. Disease-association *P* values were adjusted using the Benjamini–Hochberg procedure, and adjusted *P* values below 0.05 were considered statistically significant. Because the analysis was based on circRNA host genes, the resulting disease terms were interpreted as biological context rather than as direct evidence that individual circRNAs were causally associated with the corresponding diseases.

### 2.7 Data visualization and software

All statistical analyses and visualizations were performed in R using scripted workflows to support reproducibility. DESeq2 was used for differential-expression analysis, pROC was used for ROC analysis, and the base R generalized linear modeling framework was used for logistic regression. Figures were generated using R-based visualization packages. Analysis scripts are available from the corresponding author upon reasonable request, subject to applicable data-sharing and participant-confidentiality requirements.

## 3 Results

### 3.1 Differential expression of circRNAs in STEMI and NSTEMI

To explore which circRNAs differed between clinical groups, we first performed differential expression analysis and visualized the results using volcano plots (Figures 1A–C). In the comparison of STEMI versus controls (Figure 1A, Supplementary Table S1), several circRNAs stood out as significantly upregulated, including hsa-CHD6_000001, hsa-PTCD3_000002, hsa-PASK_000004, hsa-PAK1_000001, hsa-UBXN4_000004, hsa-STXBP3_000002, hsa-DCTN6_000002, and hsa-AGGF1_000002. The most downregulated circRNAs in this contrast were hsa-SEPT11_000030 and hsa-CENPK_000003. For NSTEMI versus controls (Figure 1B, Supplementary Table S2), the top upregulated circRNAs included hsa-CHD6_000001, hsa-LCORL_000030, hsa-PPIL4_000002, hsa-RCAN3_000002, hsa-TRAPPC9_000002, hsa-PASK_000004, hsa-STXBP3_000002, hsa-RPRD1B_000001, and hsa-RPS6KC1_000007, with hsa-TOP3A_000006 being the most strongly downregulated.

**Figure 1:**
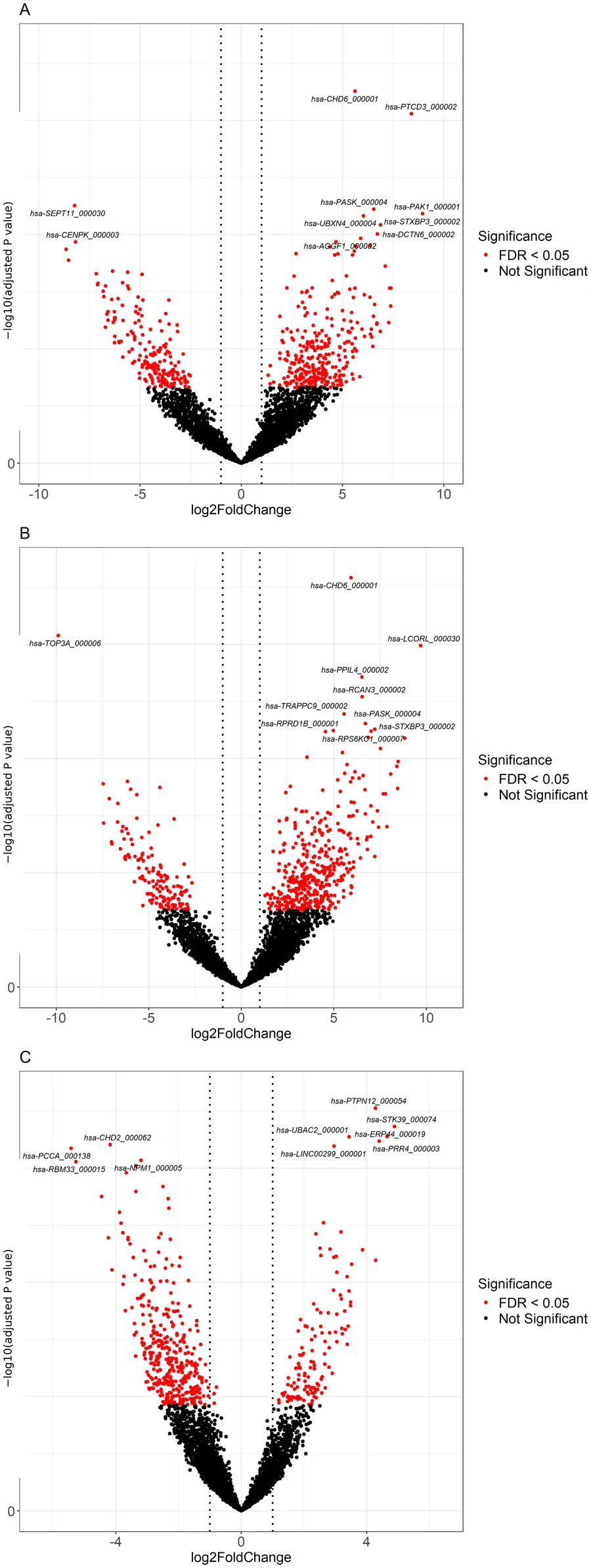
Differential expression of circRNAs across STEMI, NSTEMI, and control-group comparisons. Volcano plots show differentially expressed circular RNAs (circRNAs) in three pairwise comparisons: (**A**) STEMI versus controls, (**B**) NSTEMI versus controls, and (**C**) STEMI versus NSTEMI. Each point represents an individual circRNA. The x-axis shows the log2 fold change, and the y-axis shows the −log10 adjusted P value. Positive log2 fold-change values indicate greater circRNA abundance in the first group named in each comparison, whereas negative values indicate lower abundance in the first group. Red points represent significantly differentially expressed circRNAs at a false-discovery rate (FDR) below 0.05, whereas black points represent circRNAs that did not meet this threshold. Selected top-ranked circRNAs are labelled. The plots demonstrate differences in circulating circRNA profiles between each myocardial infarction group and controls and between STEMI and NSTEMI.

When directly comparing STEMI to NSTEMI (Figure 1C, Supplementary Table S3), 297 circRNAs were differentially expressed, indicating that the two acute MI phenotypes have distinguishable circulating circRNA profiles. The strongest STEMI-enriched circRNAs included hsa-PTPN12_000054 (log2FC = 4.28, adjusted *p* = 6.88 × 10^−7), hsa-UBAC2_000001 (log2FC = 3.44, adjusted *p* = 7.81 × 10^−7), hsa-STK39_000074 (log2FC = 4.88, adjusted *p* = 7.81 × 10^−7), hsa-ERP44_000019 (log2FC = 4.65, adjusted *p* = 7.81 × 10^−7), hsa-PRR4_000003 (log2FC = 4.39, adjusted *p* = 8.45 × 10^−7), and hsa-LINC00299_000001 (log2FC = 2.96, adjusted *p* = 8.54 × 10^−7). In contrast, hsa-CHD2_000062 (log2FC = −4.18, adjusted *p* = 8.54 × 10^−7), hsa-PCCA_000138 (log2FC = −5.43, adjusted *p* = 8.69 × 10^−7), hsa-RBM33_000015 (log2FC = −5.27, adjusted *p* = 1.60 × 10^−6), and hsa-NPM1_000005 (log2FC = −3.20, adjusted *p* = 1.60 × 10^−6) were more prevalent in NSTEMI. Together, these findings show that the STEMI versus NSTEMI contrast is not just a weaker version of the case-control comparison, but instead reveals a distinct subtype-associated circRNA pattern.

To make it easier to compare statistical significance, effect size, and how well different markers distinguish between groups, Supplementary Table S4 provides a summary of the top circRNAs in the direct comparison between STEMI and NSTEMI. The table includes log2 fold change, adjusted *p*-values, and single-marker ROC-AUC for each circRNA. A positive log2 fold change means the circRNA is more abundant in STEMI, while a negative value means it is more abundant in NSTEMI.

### 3.2 Relationship between circRNAs and host-gene expression

To determine whether circRNA changes simply mirrored changes in the corresponding host genes, we compared the log2 fold-change for each circRNA with its corresponding host gene linear transcript in all contrasts. In STEMI versus controls, there was often imperfect concordance: some circRNAs changed in parallel with their host genes, but many did not, and some even moved in the opposite direction (Supplementary Figure S1). This pattern, also observed in NSTEMI versus controls (Supplementary Figure S2), suggests that many circRNA changes are driven by post-transcriptional mechanisms such as back-splicing or altered RNA stability. In the direct STEMI versus NSTEMI comparison, discordant changes between circRNAs and their corresponding host-gene linear transcripts persist, as shown in Figure 2, further suggesting that circRNA abundance is not uniformly explained by changes in host gene expression.

**Figure 2:**
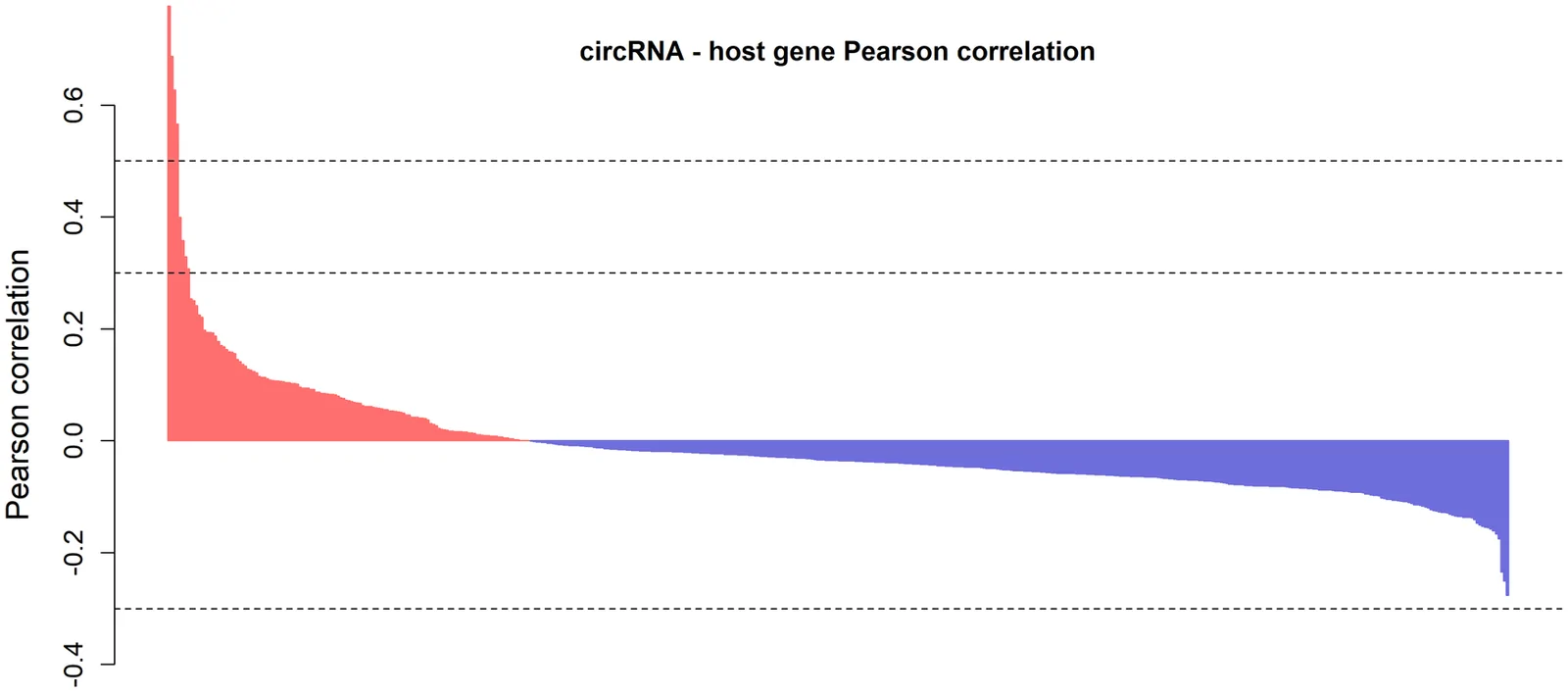
Pearson correlations between circRNA and corresponding host-gene expression. The plot shows Pearson correlation coefficients between the expression of individual circRNAs and their corresponding host-gene linear transcripts. Each bar represents one circRNA–host gene pair, and pairs are ordered from the strongest positive to the strongest negative correlation. Positive correlations are shown in red and negative correlations in blue. Most correlation coefficients are close to zero, indicating that circRNA abundance does not consistently parallel the expression of the corresponding host-gene linear transcript. The horizontal dashed lines indicate correlation-coefficient reference thresholds.

### 3.3 Identification of discriminatory circRNAs by ROC analysis

Differential-expression magnitude and statistical significance do not necessarily correspond directly to patient-level discriminatory performance. Therefore, after identifying circRNAs that were significantly differentially expressed between STEMI and NSTEMI at an FDR below 0.05, we separately assessed their individual discriminatory performance using ROC analysis.

The top-ranked circRNAs according to individual ROC-AUC were hsa-KDM1A_000054, with an AUC of 0.705 and an adjusted *P* value of 2.99 × 10⁻^3^; hsa-ITPR1_000009, with an AUC of 0.677 and an adjusted *P* value of 1.25 × 10⁻⁴; hsa-UBE2K_000030, with an AUC of 0.654 and an adjusted *P* value of 1.12 × 10⁻^3^; hsa-PPP3CC_000001, with an AUC of 0.648 and an adjusted *P* value of 1.05 × 10⁻^2^; and hsa-LINC00299_000001, with an AUC of 0.632 and an adjusted *P* value of 8.54 × 10⁻⁷. These values indicated moderate individual discriminatory performance. The complete single-marker ROC results are provided in Supplementary Table S3.

Among the individual circRNAs selected for the exploratory three-marker panel, hsa-PTPN12_000054, hsa-LINC00299_000001, and hsa-PPP3CC_000001 produced AUC values of 0.614, 0.632, and 0.648, respectively. The combined three-circRNA logistic regression model produced an apparent AUC of 0.866, while five-fold internal cross-validation yielded an AUC of 0.813 (Figure 3). These findings indicate that the combined model had greater internal discriminatory performance than each of its individual component circRNAs. However, because candidate selection and model development were conducted within the same discovery cohort, these estimates may be optimistic and require independent external validation.

**Figure 3:**
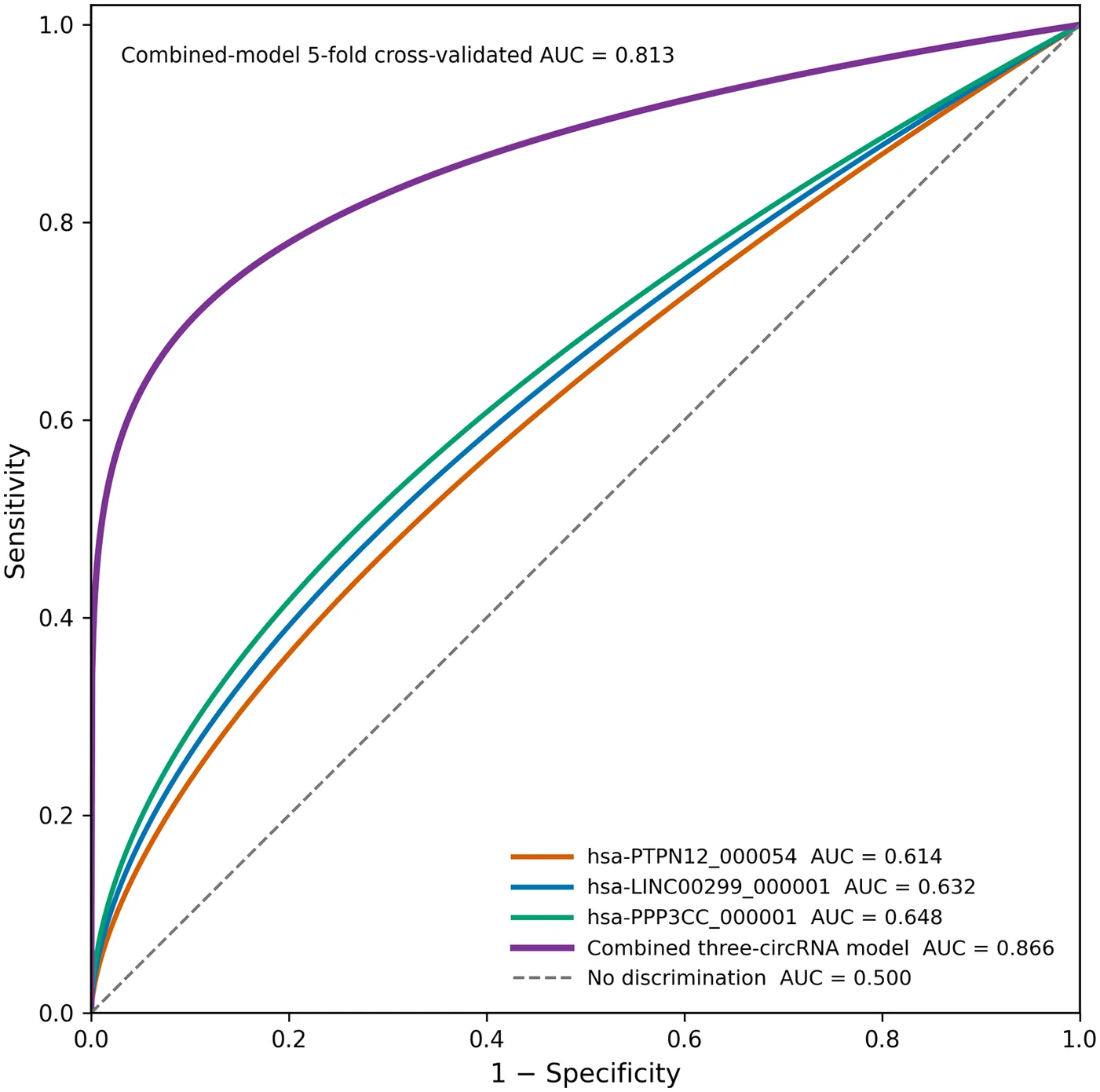
Exploratory three-circRNA model for discrimination between STEMI and NSTEMI. Receiver operating characteristic (ROC) curves show the individual discriminatory performance of hsa-PTPN12000054, hsa-LINC00299000001, and hsa-PPP3CC000001 and the apparent performance of the combined three-circRNA logistic regression model for distinguishing STEMI from NSTEMI. STEMI was treated as the positive outcome. The individual-marker areas under the curve (AUCs) were 0.614 for hsa-PTPN12000054, 0.632 for hsa-LINC00299000001, and 0.648 for hsa-PPP3CC000001. The combined model produced an apparent AUC of 0.866; five-fold internal cross-validation yielded an AUC of 0.813. The diagonal dashed line represents no-discrimination performance (AUC = 0.500). Because candidate selection and model development were conducted using the same discovery cohort, the performance estimates may be optimistic and should be considered exploratory pending independent external validation.

Comparable single-marker ROC analyses were also performed for STEMI versus controls and NSTEMI versus controls, with the leading circRNAs for these contrasts reported in Supplementary Tables S1 and S2, respectively. Overall, the ROC analyses support the identified circRNAs as exploratory candidates for future multimarker models rather than as individual stand-alone diagnostic biomarkers.

### 3.4 Gene ontology and disease-association enrichment analyses

To better understand the biological context of the dysregulated circRNAs, we performed Gene Ontology enrichment analysis using their corresponding host genes. We checked three different categories: the biological processes the genes are involved in, what their main functions are, and where in the cell they tend to be found. It's important to note that these results tell us about the usual roles of the genes where the circRNAs come from, not that the circRNAs themselves are directly controlling these processes.

Researchers looked for patterns in the genes that produce circular RNAs to better understand their biological roles. They did this separately for genes linked to circRNAs that increased and decreased in each clinical comparison. In the comparison between people with STEMI and healthy controls, the most enriched biological processes involved RNA processing, mRNA stability, chromatin organization, and DNA repair (see Figure 4). For NSTEMI versus controls, similar processes related to RNA and gene regulation were identified, but the most important categories and the genes involved were somewhat different from the STEMI group (Figure 5). When directly comparing STEMI to NSTEMI, the enriched processes included intracellular transport, protein localization, nucleocytoplasmic transport, chromatin organization, and chromatin remodeling (Supplementary Figure S5 & S6). Because these analyses focus on the genes that host circRNAs, the results offer useful biological context. However, they should not be taken as direct evidence of what circRNAs actually do in these processes.

**Figure 4:**
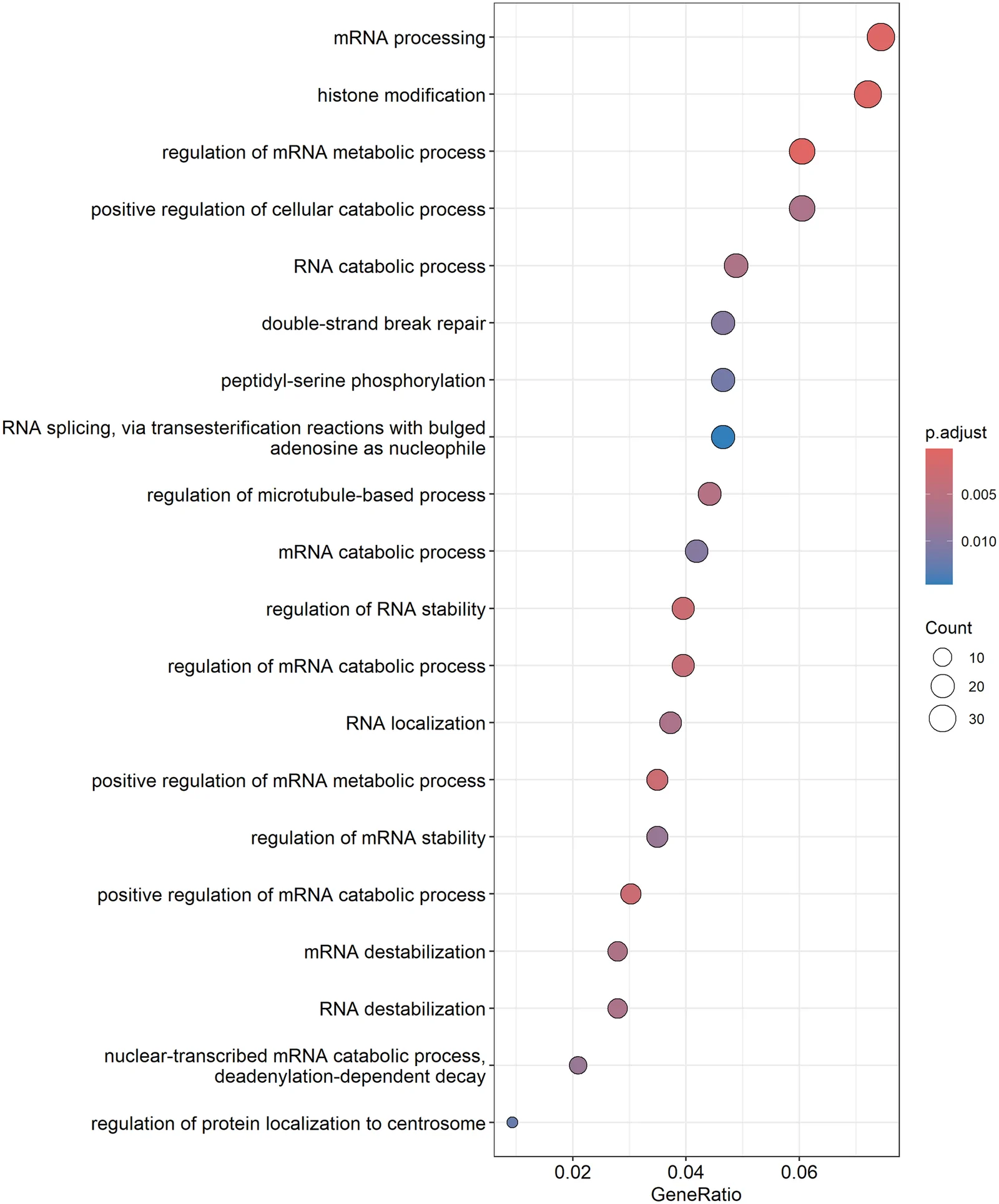
Gene Ontology biological-process enrichment of circRNA host genes in STEMI versus controls. Gene Ontology Biological Process over-representation analysis was performed using the host genes of circRNAs [upregulated/downregulated] in STEMI relative to controls. The dot plot shows the most significantly enriched biological-process terms. The x-axis represents the gene ratio, dot size represents the number of host genes contributing to each term, and dot colour represents the Benjamini-Hochberg-adjusted *P* value. The enrichment analysis provides biological context for the host genes of dysregulated circRNAs but does not establish the molecular functions or causal effects of the circRNAs themselves.

**Figure 5:**
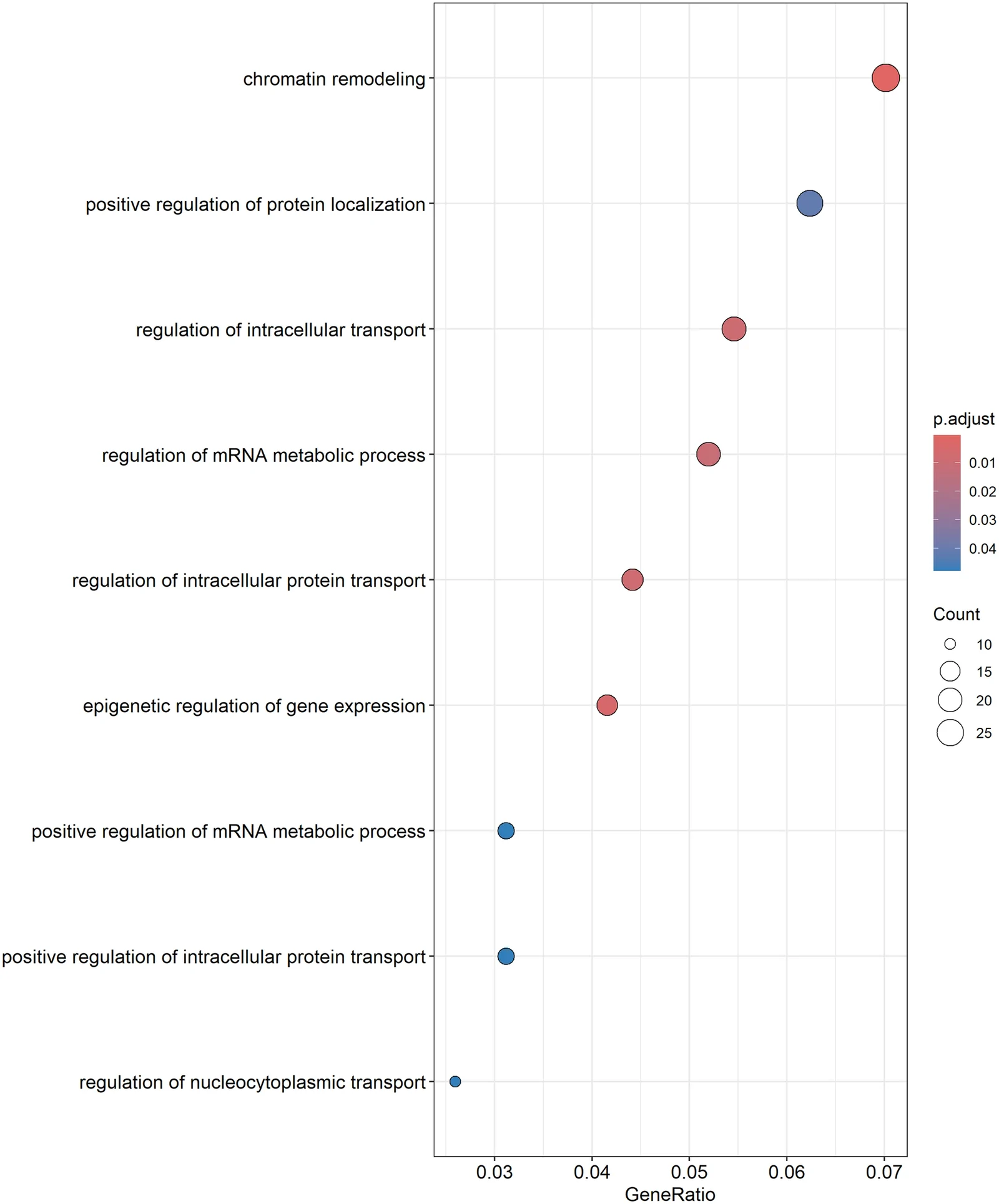
Gene Ontology biological-process enrichment of circRNA host genes in NSTEMI versus controls. Gene Ontology Biological Process over-representation analysis was performed using the host genes of circRNAs [upregulated/downregulated] in NSTEMI relative to controls. The dot plot shows the most significantly enriched biological-process terms. The x-axis represents the gene ratio, dot size represents the number of host genes contributing to each term, and dot color represents the Benjamini–Hochberg-adjusted *P*-value. The enrichment analysis provides biological context for the host genes of dysregulated circRNAs but does not establish the molecular functions or causal effects of the circRNAs themselves.

Additional enrichment analyses were conducted separately for the host genes of upregulated and downregulated circRNAs. In each pair of Supplementary Figures cited below, the first figure presents the enrichment dot plot, while the second presents the corresponding category-network plot. Biological Process results for the host genes of downregulated circRNAs in the STEMI-versus-NSTEMI comparison are presented in Supplementary Figures S4 and S5.

Molecular Function results for the host genes of downregulated circRNAs are presented for STEMI versus controls in Supplementary Figures S6 and S7, NSTEMI versus controls in Supplementary Figures S8 and S9, and NSTEMI versus STEMI in Supplementary Figures S10 and S11. Cellular Component results for the host genes of downregulated circRNAs in the STEMI-versus-control comparison are presented in Supplementary Figures S12 and S13. This analysis identified terms associated with nuclear specks and spliceosomal complexes, which are involved in RNA processing and splicing.

For the host genes of upregulated circRNAs, Biological Process results are presented for STEMI versus controls in Supplementary Figures S14 and S15, NSTEMI versus controls in Supplementary Figures S16 and S17, and STEMI versus NSTEMI in Supplementary Figures S18 and S19. Molecular Function results for the same comparisons are presented in Supplementary Figures S20–S25, respectively. Cellular Component results are presented for STEMI versus controls in Supplementary Figures S26 and S27 and NSTEMI versus controls in Supplementary Figures S28 and S29.

Overall, the Gene Ontology results indicate that the host genes of dysregulated circRNAs are associated with biological processes involving RNA metabolism, chromatin regulation, intracellular transport, and cellular stress responses. However, because the analyses were based on circRNA host genes rather than experimentally established molecular targets of the circRNAs, these findings provide biological context but do not establish the molecular functions or causal roles of individual circRNAs.

### 3.5 Disease association analysis

Finally, we used DisGeNET enrichment to connect the host genes of dysregulated circRNAs with known disease annotations. In the STEMI versus NSTEMI comparison, disease terms were strongly linked to cardiovascular conditions, including coronary artery disease, acute coronary syndrome, myocardial infarction, and broader cardiovascular disease. These pathway patterns were consistent across various enrichment analyses, suggesting that the circRNA changes, particularly those differentiating STEMI from NSTEMI, reflect distinct biological programs rather than random variation. It is important to note that DisGeNET results are based on host genes and therefore should be interpreted as biological context rather than direct evidence that each circRNA itself is causally involved in disease.

## 4 Discussion

To our knowledge, this work is among the first to directly compare the plasma circRNA landscape between STEMI and NSTEMI patients in a real-world clinical setting. What stood out immediately was the sheer number, 297, of circRNAs with differential expression between these two forms of heart attack, even though both represent acute myocardial infarction. This points to a surprisingly rich layer of molecular diversity that goes beyond what we can see on an ECG or in a troponin test.

Several circRNAs consistently stood out in our STEMI versus NSTEMI comparisons. For STEMI, hsa-PTPN12_000054, hsa-UBAC2_000001, hsa-STK39_000074, hsa-ERP44_000019, hsa-PRR4_000003, and hsa-LINC00299_000001 were especially prominent, while hsa-CHD2_000062, hsa-PCCA_000138, hsa-RBM33_000015, and hsa-NPM1_000005 were more abundant in NSTEMI samples. Importantly, these are not just generic markers of myocardial injury. Because both STEMI and NSTEMI patients experience inflammation, cell death, and systemic stress, the circRNAs that still differ between them may reflect biology unique to each subtype.

When we looked at how well these circRNAs could actually distinguish STEMI from NSTEMI, the top performers (like hsa-KDM1A_000054, hsa-ITPR1_000009, hsa-UBE2K_000030, hsa-PPP3CC_000001, and hsa-LINC00299_000001) yielded ROC-AUC values in the 0.63–0.71 range. That's promising, but it also tells us that no single circRNA is likely to replace conventional diagnostic tools. Instead, these findings suggest that circRNAs could add value as part of a more nuanced, multi-marker approach, perhaps in combination with troponins, ECGs, imaging, and clinical scores.

It's worth emphasizing that our findings add weight to a principle that's gained traction in biomarker research: the best diagnostic strategies often come from combining different types of molecular signals, rather than betting everything on a single marker. In cardiology, this isn't a new idea—many studies have tested multi-marker panels to sharpen early diagnosis and improve risk assessment in acute myocardial infarction (16, 17). The story is similar when it comes to non-coding RNAs: microRNA panels, for example, have shown promise for heart attack detection (18).

Although no individual circRNA showed sufficiently high discrimination to function as a stand-alone marker, the improved internal performance of the exploratory three-circRNA model supports the potential value of combining complementary circRNA signals. Nevertheless, the panel was selected and evaluated within the same discovery cohort, and its performance may therefore be optimistic. A prespecified model with locked markers and coefficients should be evaluated in an independent cohort before any clinical conclusions are drawn.

Our study also joins a growing body of research suggesting circRNAs are genuine players in cardiovascular disease biology. Previous work has linked circRNAs such as MICRA and circZNF609 to cardiac remodeling and repair after MI (19, 20). Other papers have described circRNAs involved in atherosclerosis and heart failure (21, 22). Yet, the top candidates we identified in the STEMI/NSTEMI comparison, like hsa-PTPN12_000054, hsa-LINC00299_000001, hsa-KDM1A_000054, and hsa-ITPR1_000009, do not simply recapitulate those prior findings, likely due to differences in patient populations, timing, or analytical methods.

A striking observation from our data is that circRNA changes often did not parallel changes in their host gene's mRNA. Sometimes the directions were even opposite, hinting that circRNAs are not just byproducts of host gene transcription but may be independently regulated, by back-splicing, altered stability, or selective release into plasma (23, 24).

When we looked at the genes that give rise to these circRNAs, we found they were mostly involved in important cell activities like processing RNA, keeping mRNA stable, managing how DNA is packed and unpacked, moving molecules in and out of the cell nucleus, and repairing damaged DNA. These kinds of processes make sense, since they play big roles when the heart is under stress from lack of oxygen, inflammation, or injury (25, 26). We also noticed that many of these genes are linked to adding methyl groups to molecules, and are found in specific cell parts like nuclear specks and spliceosomal complexes. This fits well with what other studies have reported—circRNAs have been connected to inflammation in the heart, new blood vessel growth, programmed cell death, scarring, and damage from reduced blood flow (27, 28). Still, it's important to be careful with these results. Our analysis is based on the genes where the circRNAs come from, not on direct experiments showing what the circRNAs themselves actually do. Therefore, these findings help us understand the possible biological context, but they don't prove exactly how each circRNA works or whether it causes any of these effects.

Clinically, the take-home message is not that a single circRNA is ready for prime time as a diagnostic marker. Rather, circRNA profiling may one day complement established tools, helping to refine risk stratification and identify biological subtypes that could matter for prognosis or therapy (28, 29). The moderate AUCs we observed make it clear that any clinical application will require independent validation in larger, diverse cohorts, as well as integration with existing risk models.

There are several limitations to this study. First, the analysis was conducted entirely within a discovery RNA-sequencing cohort, and neither the leading circRNA candidates nor the exploratory three-circRNA model was validated using RT-qPCR, digital PCR, or an independent external cohort. The reported effect sizes and AUC estimates should therefore be considered preliminary and hypothesis-generating. Furthermore, internal cross-validation cannot fully eliminate the optimism introduced when feature selection and model development are performed within the same discovery cohort. Accordingly, although the cross-validated AUC provides an estimate of internal performance, it should not be interpreted as an unbiased estimate of the model's performance in an independent population.

Age matching was not optimal, although this is less likely to affect the direct STEMI-versus-NSTEMI comparison than the comparisons with controls. Residual confounding also remains possible because diabetes mellitus, hypertension, dyslipidemia, renal function, medication exposure, time from symptom onset to sampling, infarct size, and reperfusion treatment may influence circulating RNA profiles. These variables were unavailable or insufficiently complete for reliable inclusion in the primary model. In addition, circulating plasma circRNAs may originate from multiple tissues and cannot be assumed to be cardiac-specific. The observed RNA-level associations also do not establish that the identified circRNAs have causal roles in myocardial infarction.

Future studies should confirm the back-splice junctions of the leading candidates using divergent-primer RT-qPCR or digital PCR and evaluate a prespecified, locked three-circRNA model in an independent, clinically representative cohort. Larger studies with comprehensive clinical phenotyping will also be required to assess potential confounding, examine temporal changes through longitudinal sampling, and determine whether the identified circRNAs provide incremental diagnostic or prognostic value beyond established clinical variables, ECG findings, and cardiac biomarkers. Mechanistic studies will be needed to establish whether these circRNAs actively contribute to disease processes or serve primarily as circulating biomarkers.

## 5 Conclusion

In conclusion, In this exploratory plasma RNA-sequencing study, STEMI and NSTEMI were associated with distinguishable circulating circRNA expression patterns. Several circRNAs showed significant subtype-associated differences, although individual-marker discrimination was modest. An exploratory three-circRNA model showed improved internal discrimination, but its performance may be optimistic because feature selection and model development were conducted in the same discovery cohort. By comparing our findings to both established and emerging circRNA signatures in cardiovascular disease, and by analyzing their relationship with host gene expression, we provide new insights into the molecular underpinnings of acute myocardial injury. Technical confirmation and independent external validation are required before the identified circRNAs can be considered for clinical biomarker applications.

## Data Availability

The datasets presented in this study can be found in online repositories. The names of the repository/repositories and accession number(s) can be found below: https://www.ebi.ac.uk/ena, PRJEB104626.
