# Supplementary material for: Distinct circular RNA signatures distinguish STEMI from NSTEMI in acute myocardial infarction
Source: Front Cardiovasc Med. 2026 Sep 25;13:1913930. doi: 10.3389/fcvm.2026.1913930 (PMC13649466; doi:10.3389/fcvm.2026.1913930)
Supplement: Supplementary file 1 [file Datasheet1.pdf]

Supplementary Material

1 Supplementary Data

None

2 Supplementary Figures and Tables

2.1 Supplementary Figures

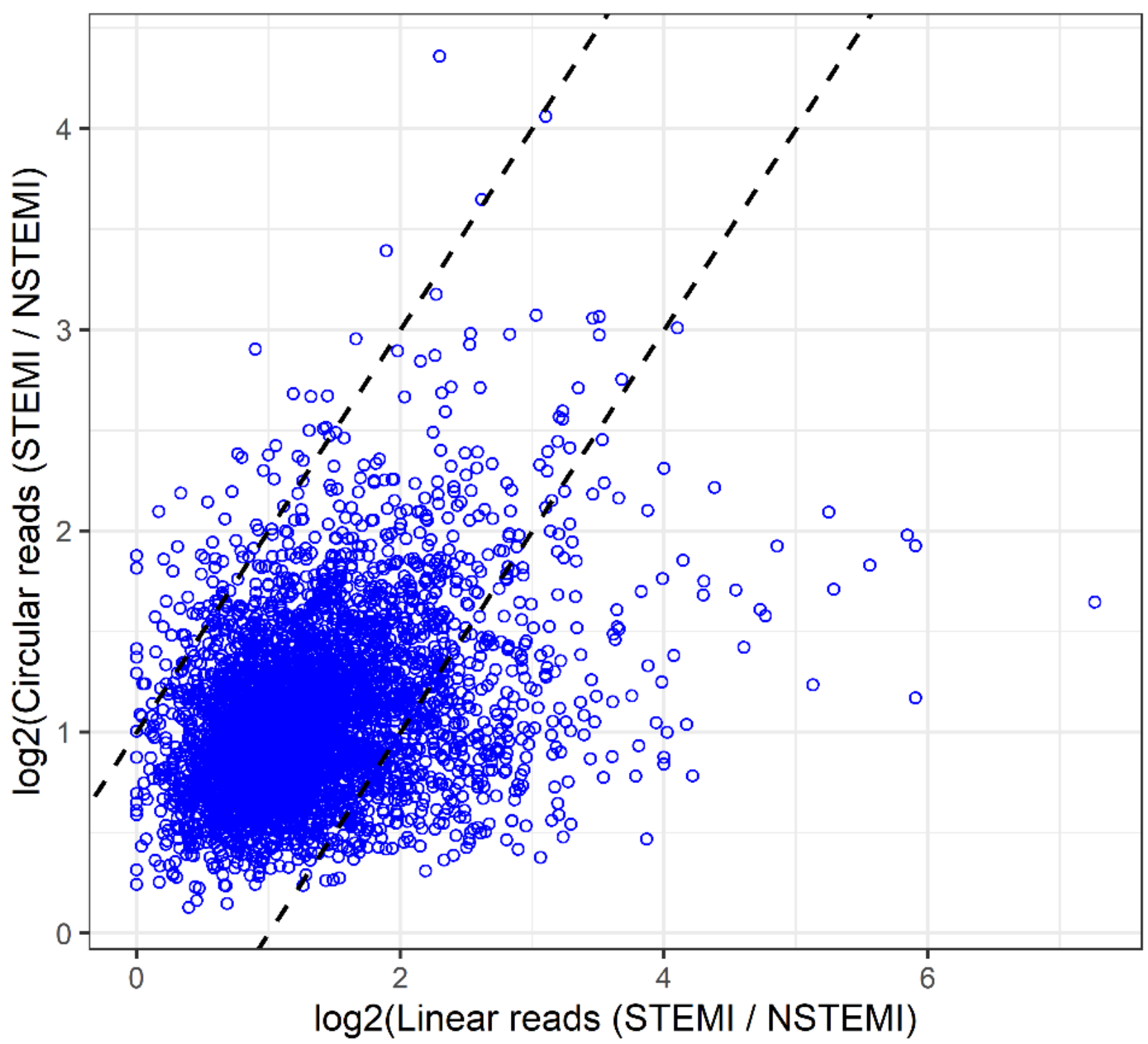

Supplementary Figure S1. The scatter plot showing the relationship between circular RNA and corresponding linear RNA expression changes in the STEMI versus NSTEMI comparison. Each point represents a circRNA-host gene pair, allowing assessment of whether circRNA changes mirror or diverge from host-gene linear transcript changes.

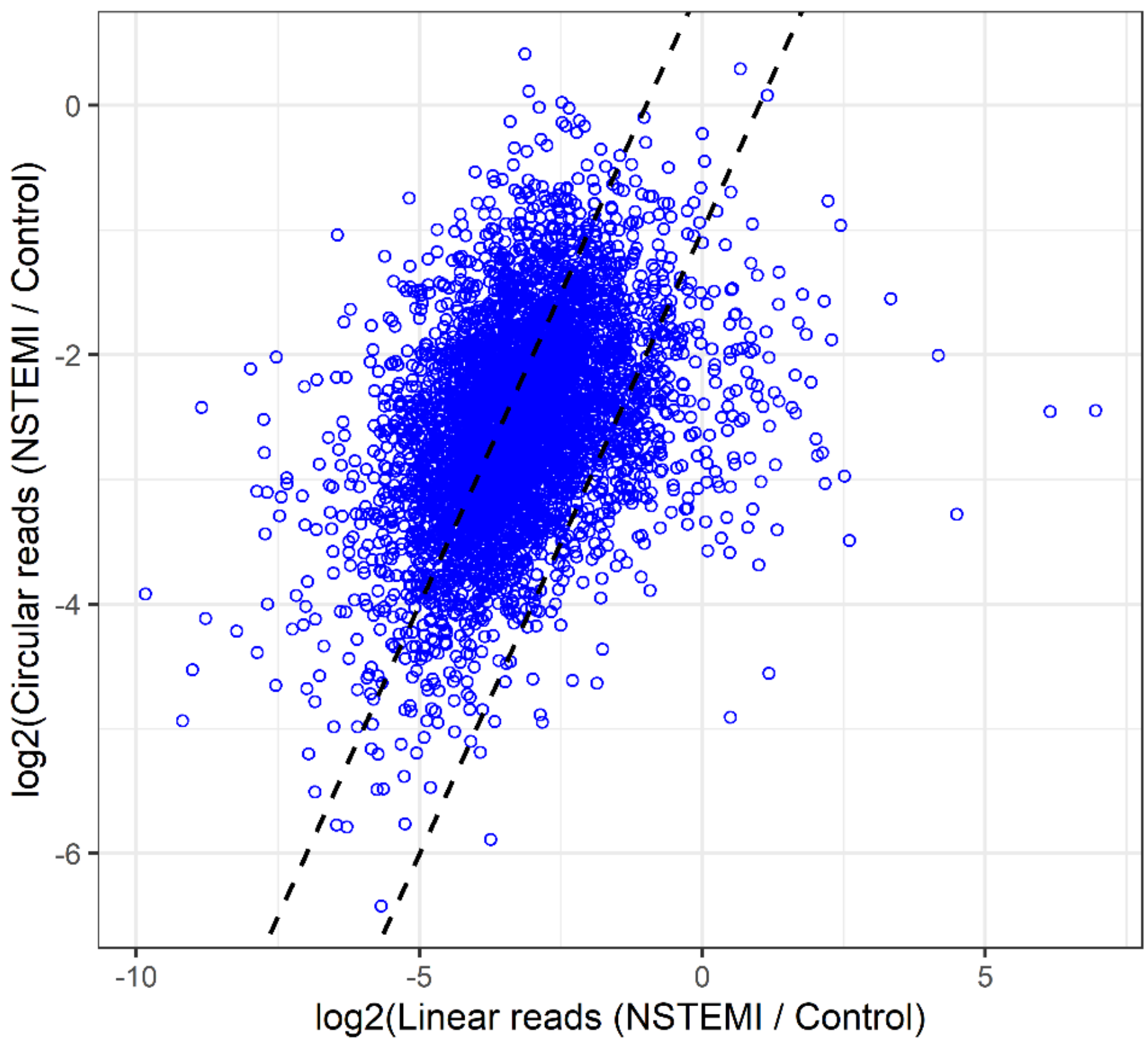

Supplementary Figure S2. Scatter plot shows the relationship between circular RNA and corresponding linear RNA expression changes in NSTEMI patients compared with control subjects. The plot illustrates the extent to which NSTEMI-associated circRNA changes correspond to, or occur independently from, changes in host-gene linear RNA expression.

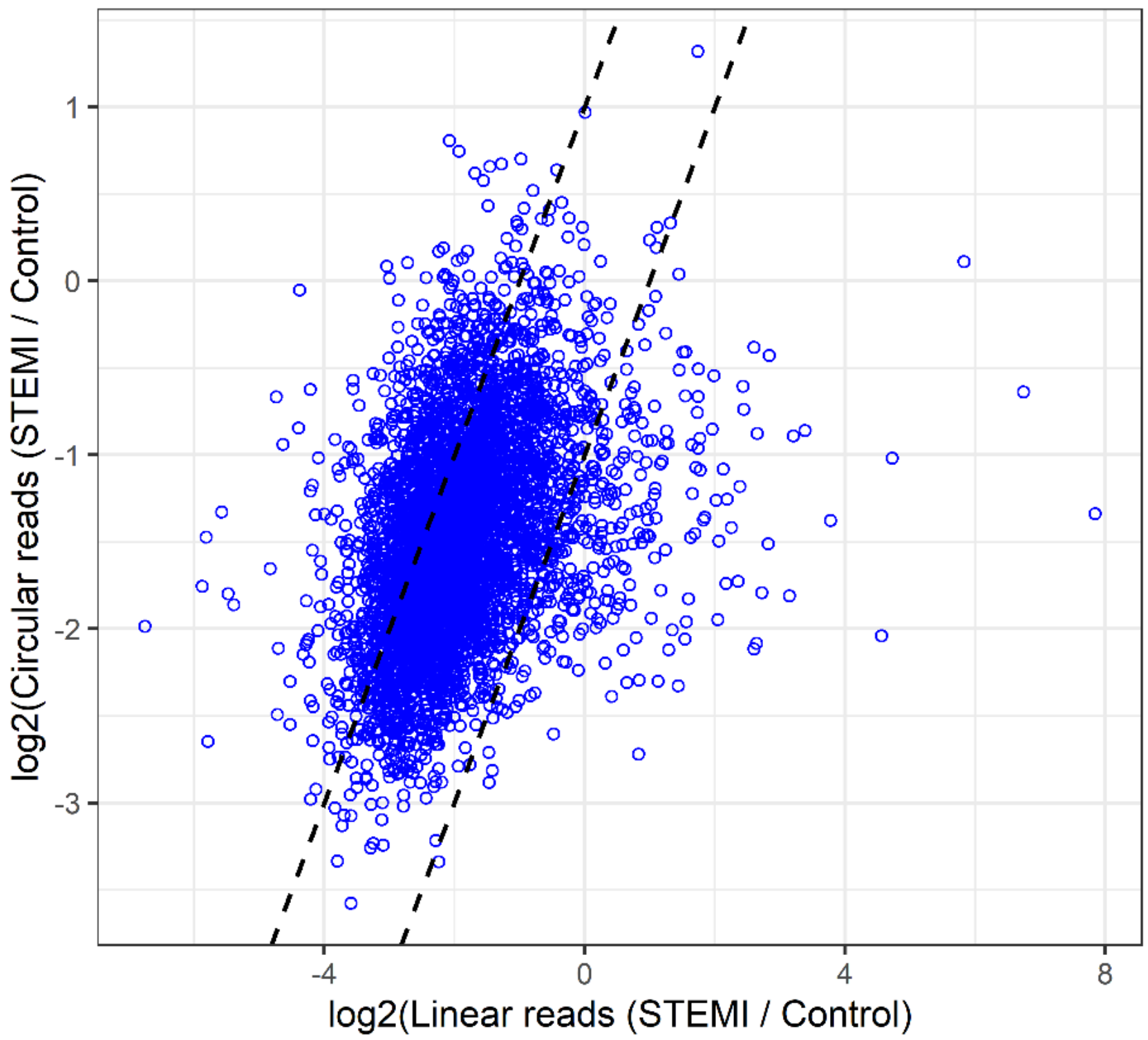

Supplementary Figure S3. The scatter plot shows the relationship between circular RNA and corresponding linear RNA expression changes in STEMI patients compared with control subjects. The distribution highlights concordant and discordant patterns between circRNA abundance and host-gene linear transcript expression.

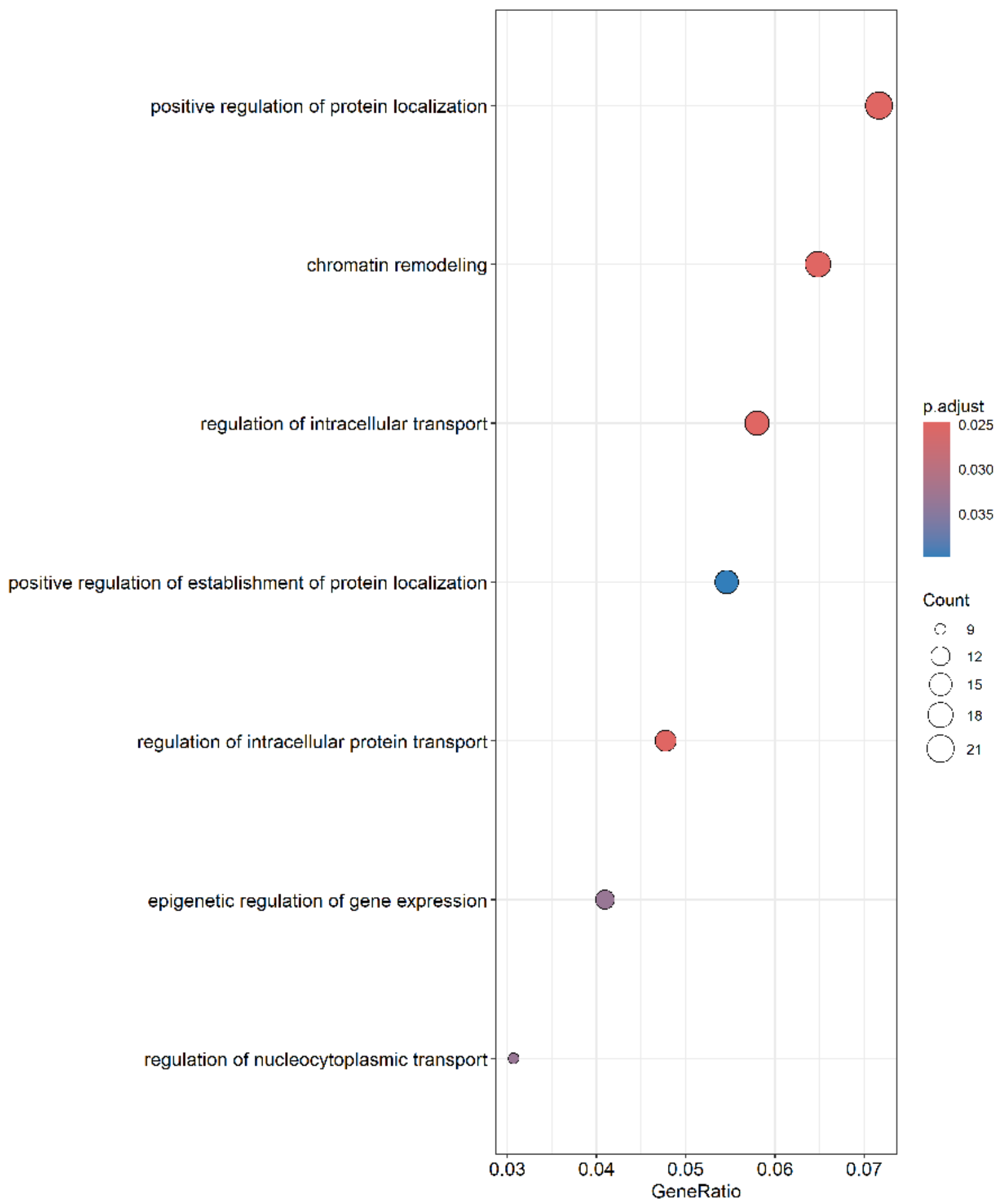

Supplementary Figure S4. Gene Ontology biological process enrichment dot plot showing terms significantly associated with host genes of downregulated circRNAs in the STEMI vs NSTEMI comparison. The plot summarizes enriched functional categories and provides pathway-level context for circRNA-associated changes in this contrast.

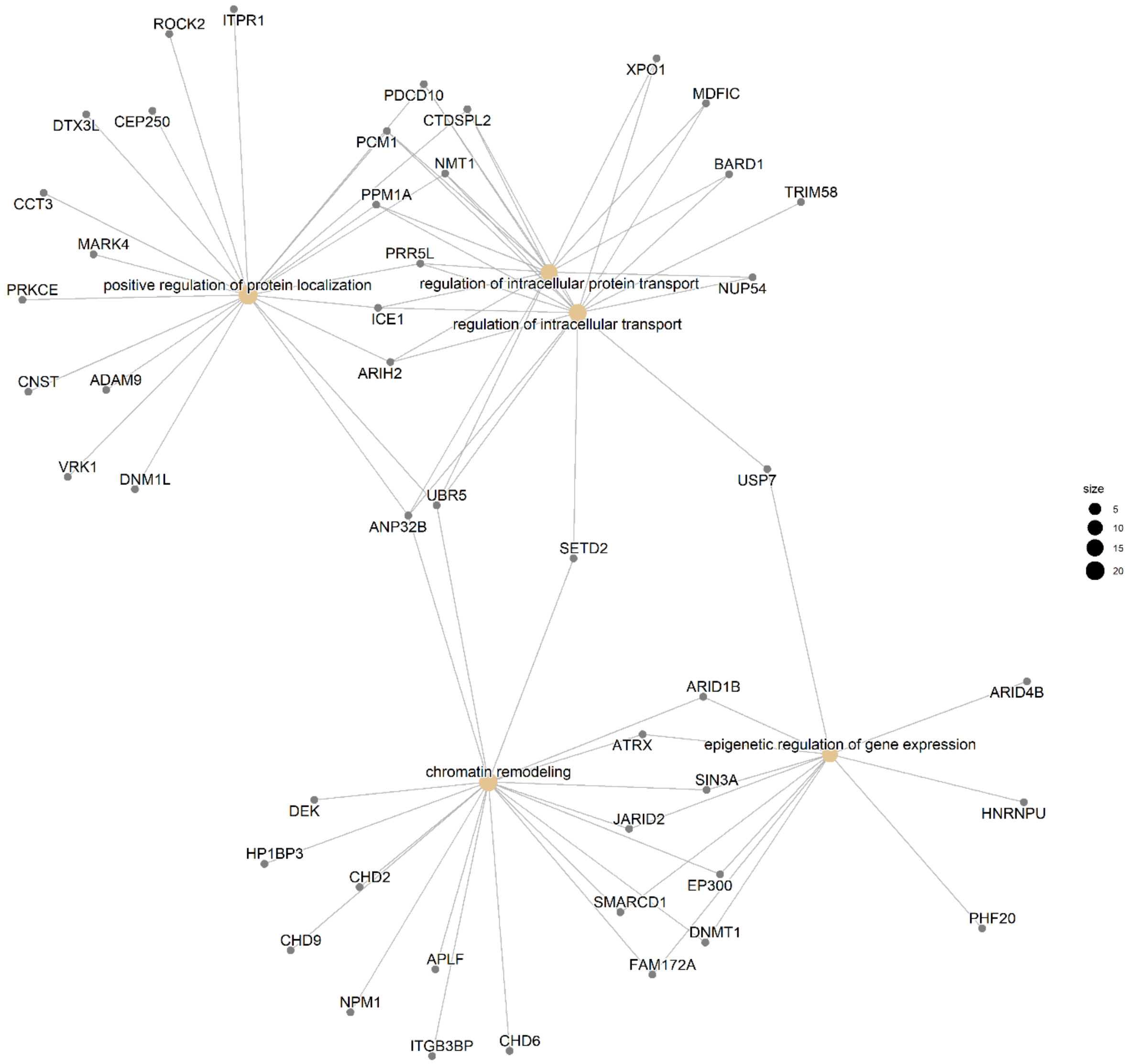

Supplementary Figure S5. Gene Ontology biological process category network plot showing relationships among enriched terms significantly associated with host genes of downregulated circRNAs in the STEMI vs NSTEMI comparison. Connected terms share contributing genes, highlighting coordinated functional modules linked to circRNA-associated changes.

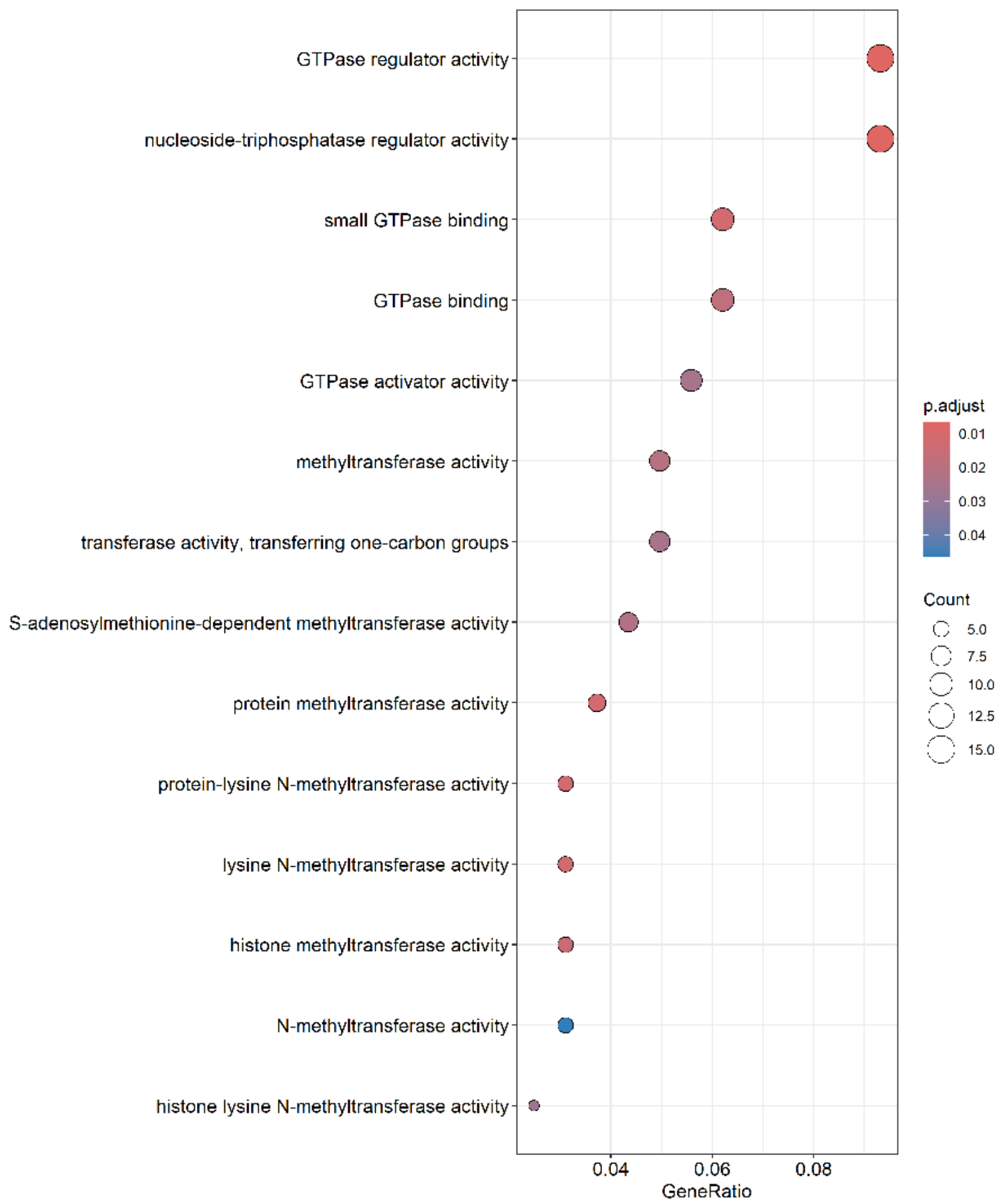

Supplementary Figure S6. Gene Ontology molecular function enrichment dot plot showing terms significantly associated with host genes of downregulated circRNAs in the STEMI vs Control comparison. The plot summarizes enriched functional categories and provides pathway-level context for circRNA-associated changes in this contrast.

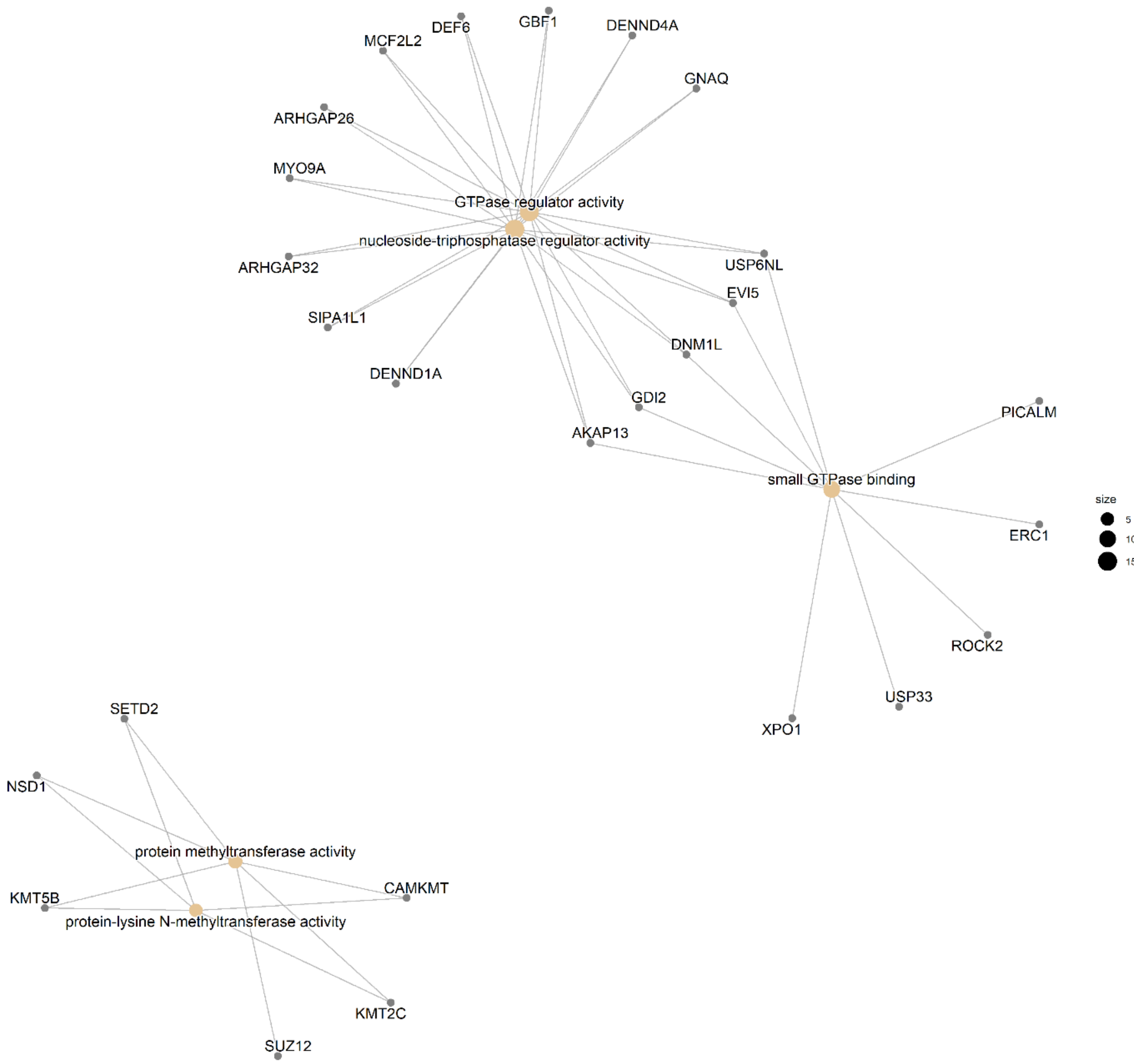

Supplementary Figure S7. Gene Ontology molecular function category network plot showing relationships among enriched terms significantly associated with host genes of downregulated circRNAs in the STEMI vs Control comparison. Connected terms share contributing genes, highlighting coordinated functional modules linked to circRNA-associated changes.

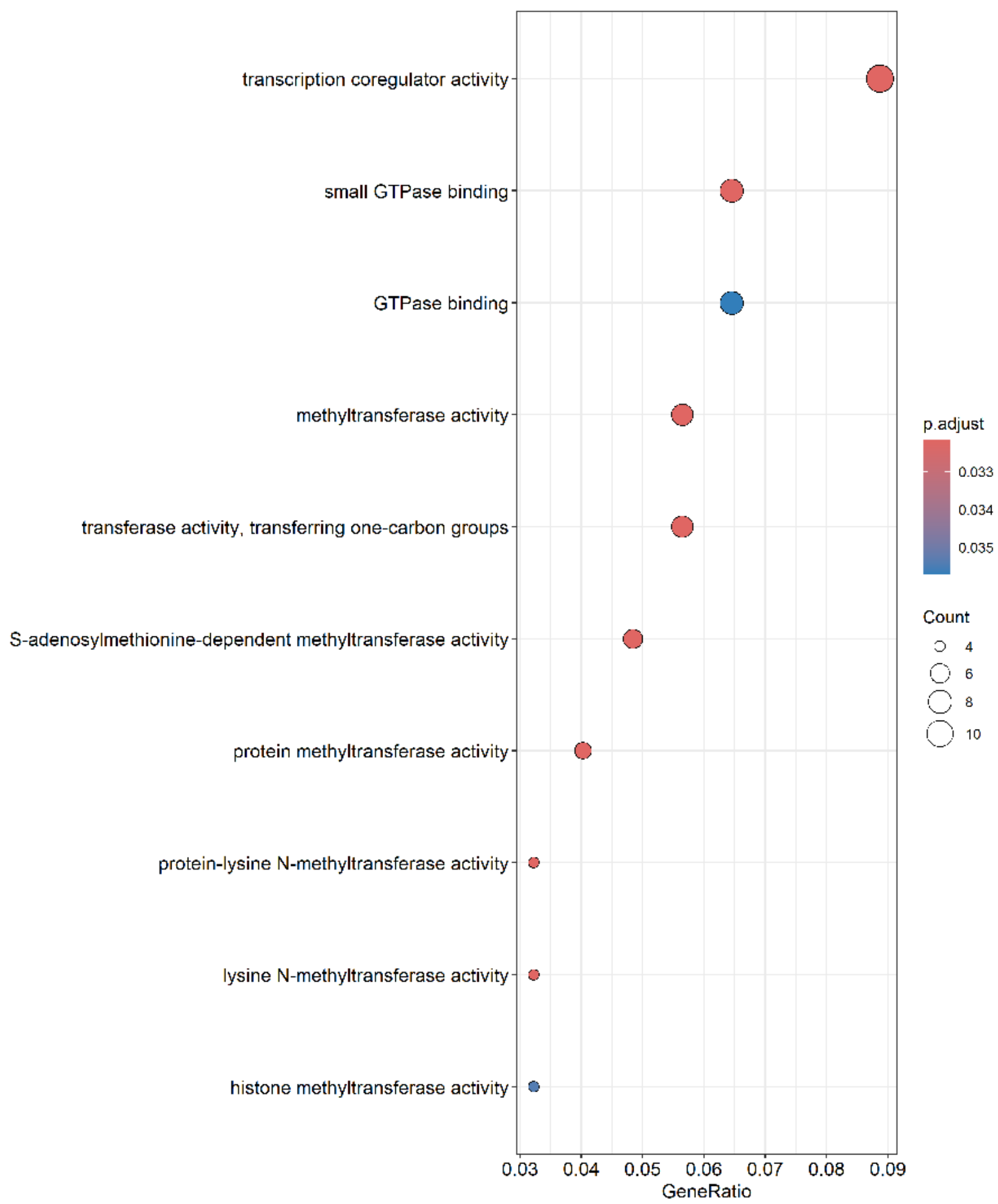

Supplementary Figure S8. Gene Ontology molecular function enrichment dot plot showing terms significantly associated with host genes of downregulated circRNAs in the NSTEMI vs Control comparison. The plot summarizes enriched functional categories and provides pathway-level context for circRNA-associated changes in this contrast.

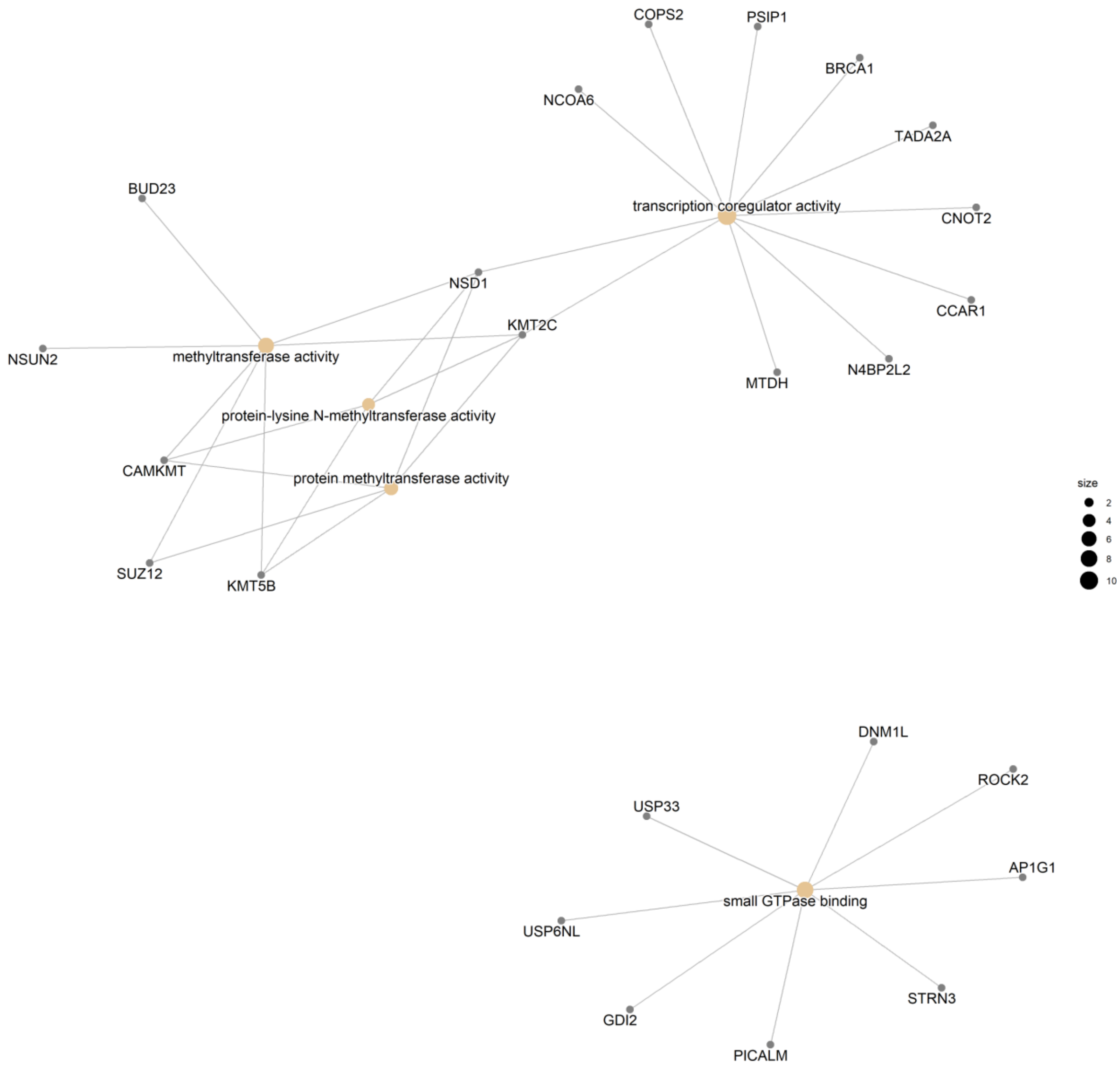

Supplementary Figure S9. Gene Ontology molecular function category network plot showing relationships among enriched terms significantly associated with host genes of downregulated circRNAs in the NSTEMI vs Control comparison. Connected terms share contributing genes, highlighting coordinated functional modules linked to circRNA-associated changes.

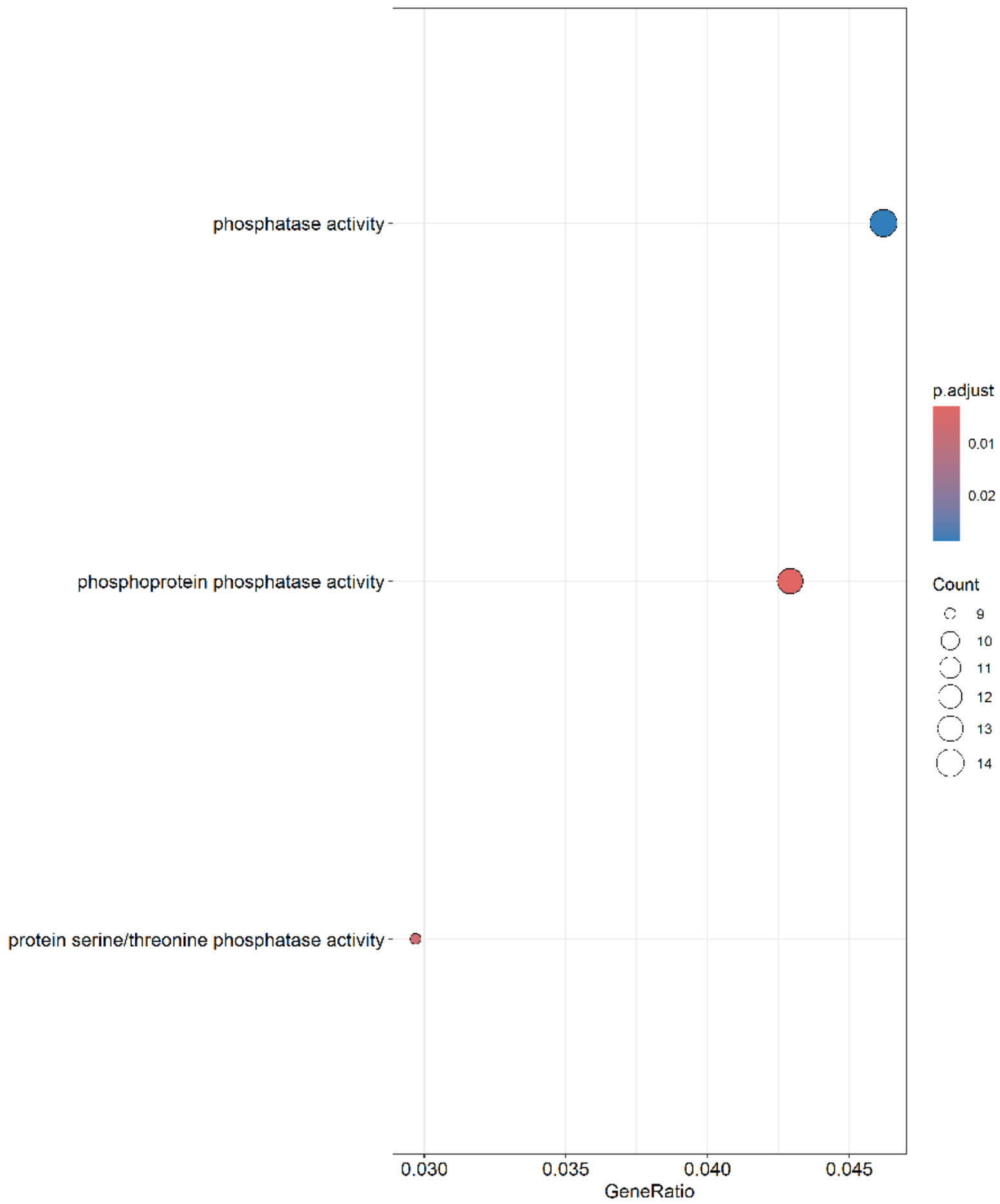

Supplementary Figure S10. Gene Ontology molecular function enrichment dot plot showing terms significantly associated with host genes of downregulated circRNAs in the NSTEMI vs STEMI comparison. The plot summarizes enriched functional categories and provides pathway-level context for circRNA-associated changes in this contrast.

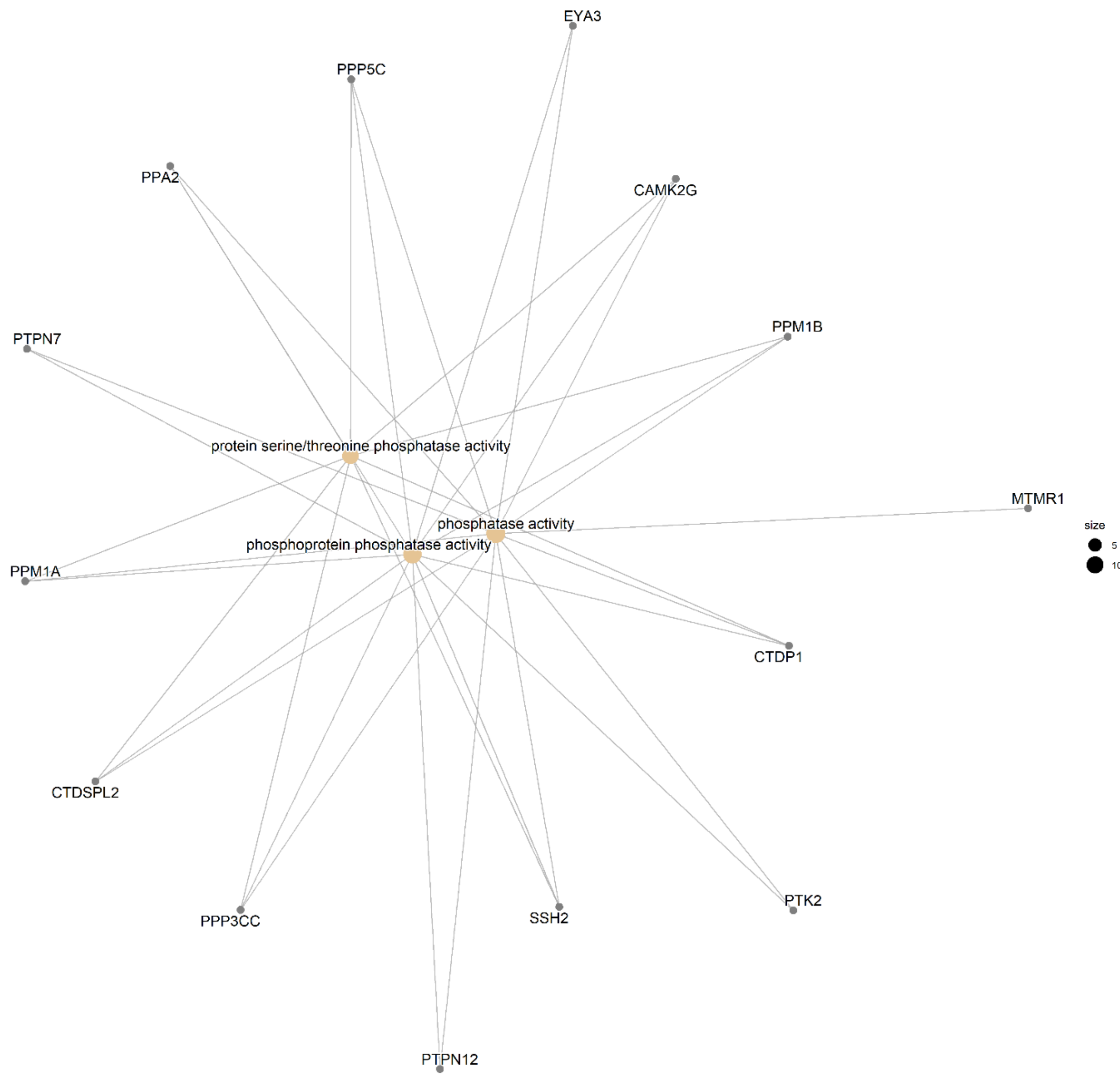

Supplementary Figure S11. Gene Ontology molecular function category network plot showing relationships among enriched terms significantly associated with host genes of downregulated circRNAs in the NSTEMI vs STEMI comparison. Connected terms share contributing genes, highlighting coordinated functional modules linked to circRNA-associated changes.

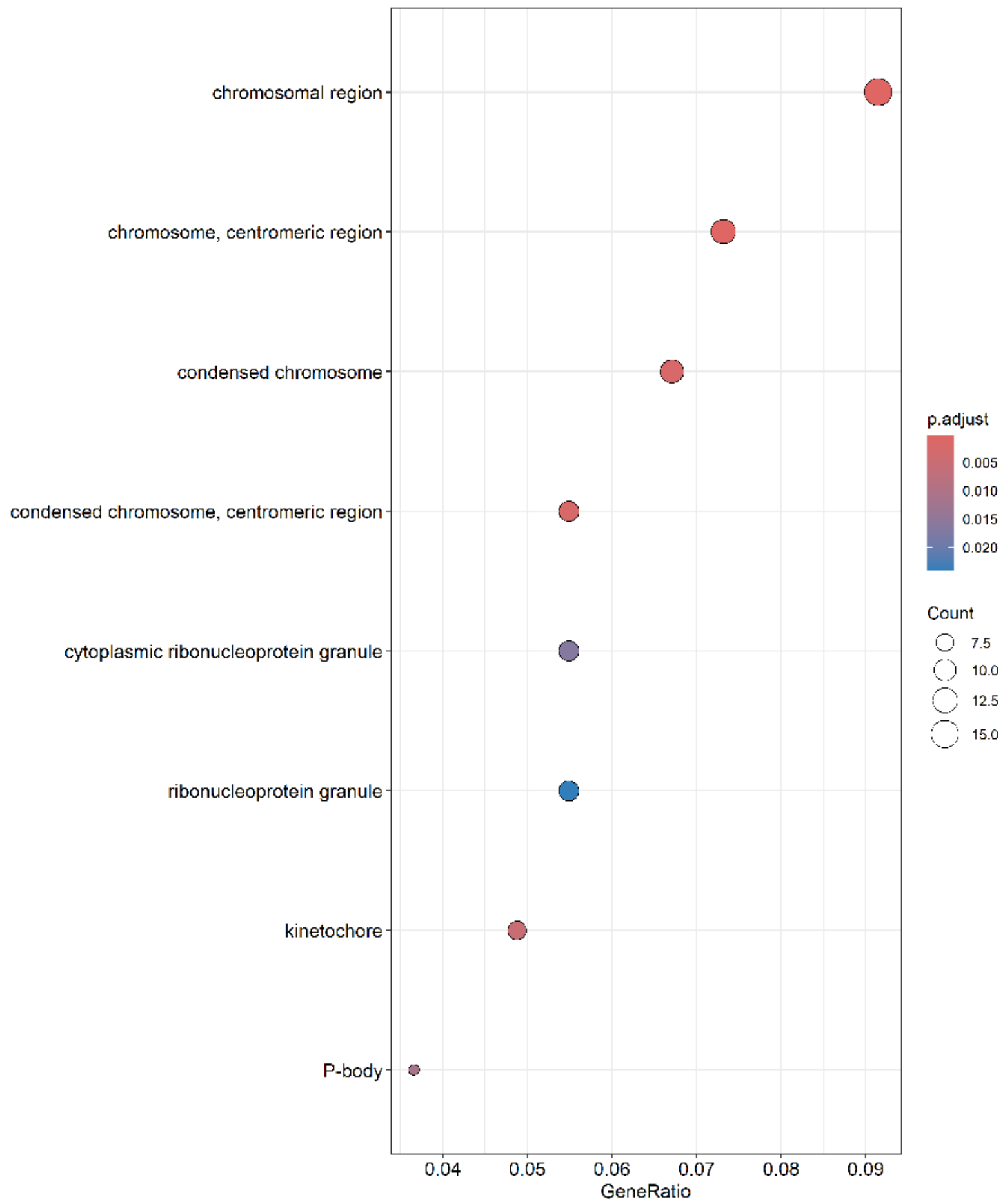

Supplementary Figure S12. Gene Ontology cellular component enrichment dot plot showing terms significantly associated with host genes of downregulated circRNAs in the STEMI vs Control comparison. The plot summarizes enriched functional categories and provides pathway-level context for circRNA-associated changes in this contrast.

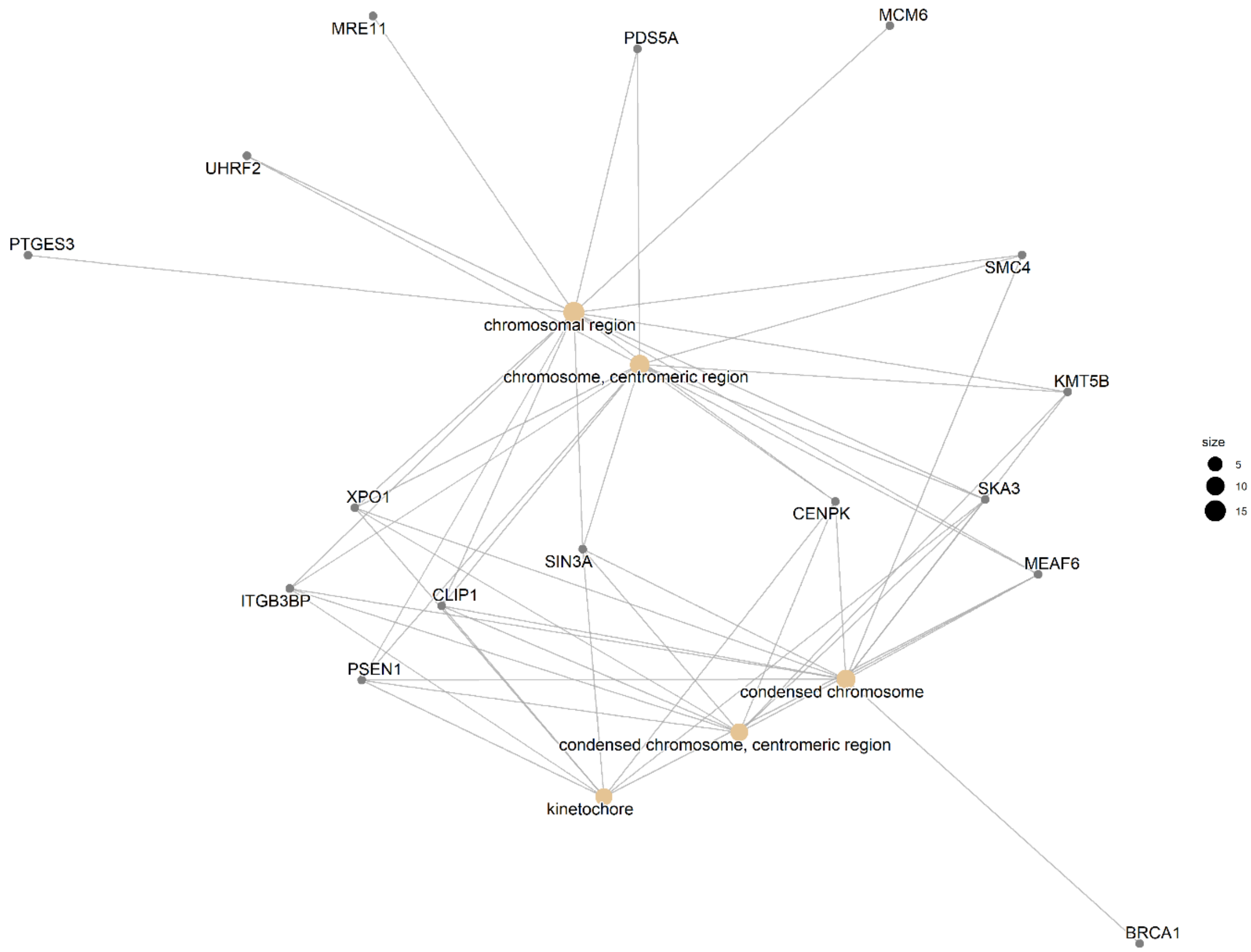

Supplementary Figure S13. Gene Ontology cellular component category network plot showing relationships among enriched terms significantly associated with host genes of downregulated circRNAs in the STEMI vs Control comparison. Connected terms share contributing genes, highlighting coordinated functional modules linked to circRNA-associated changes.

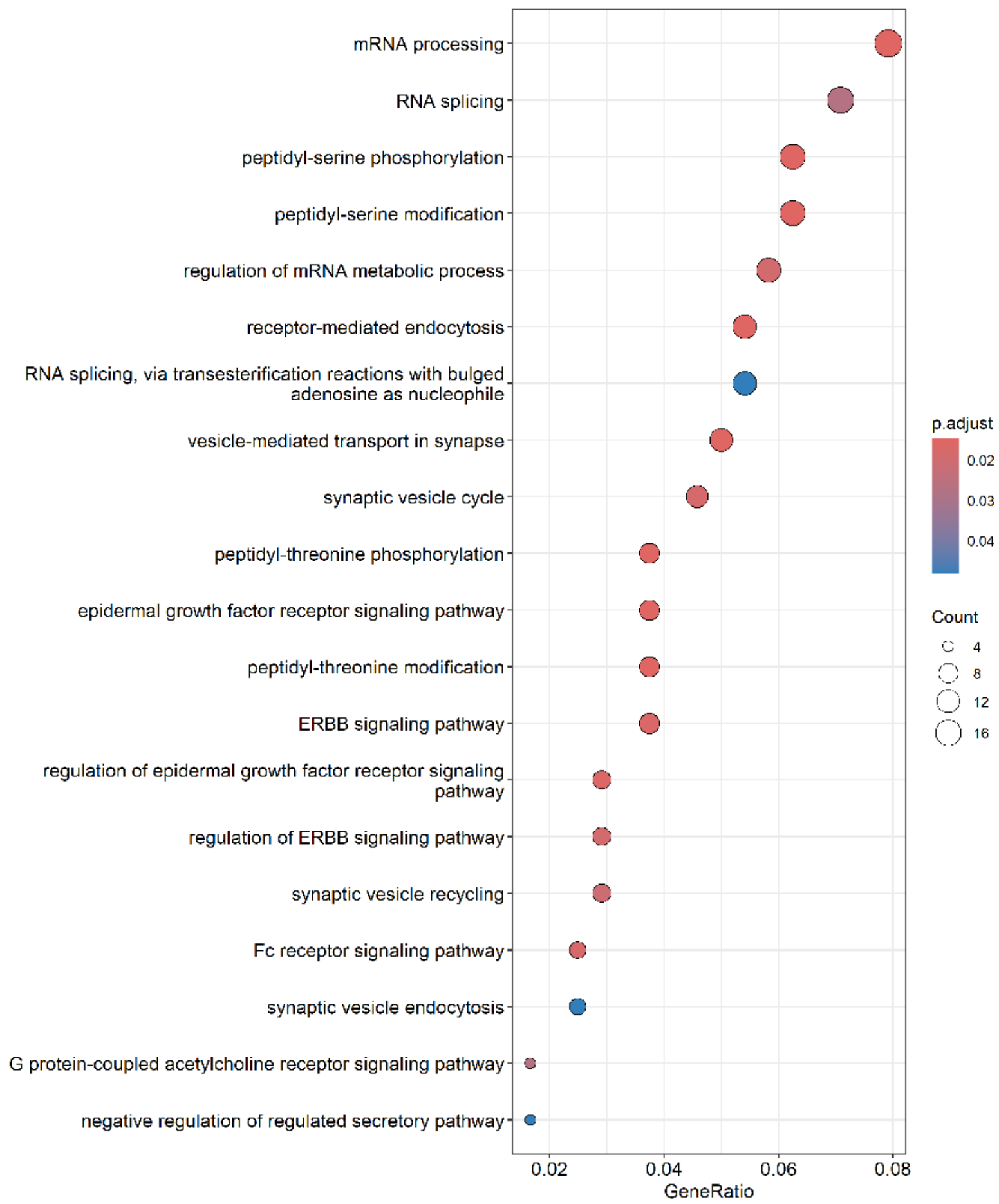

Supplementary Figure S14. Gene Ontology biological process enrichment dot plot showing terms significantly associated with host genes of upregulated circRNAs in the STEMI vs Control comparison. The plot summarizes enriched functional categories and provides pathway-level context for circRNA-associated changes in this contrast.

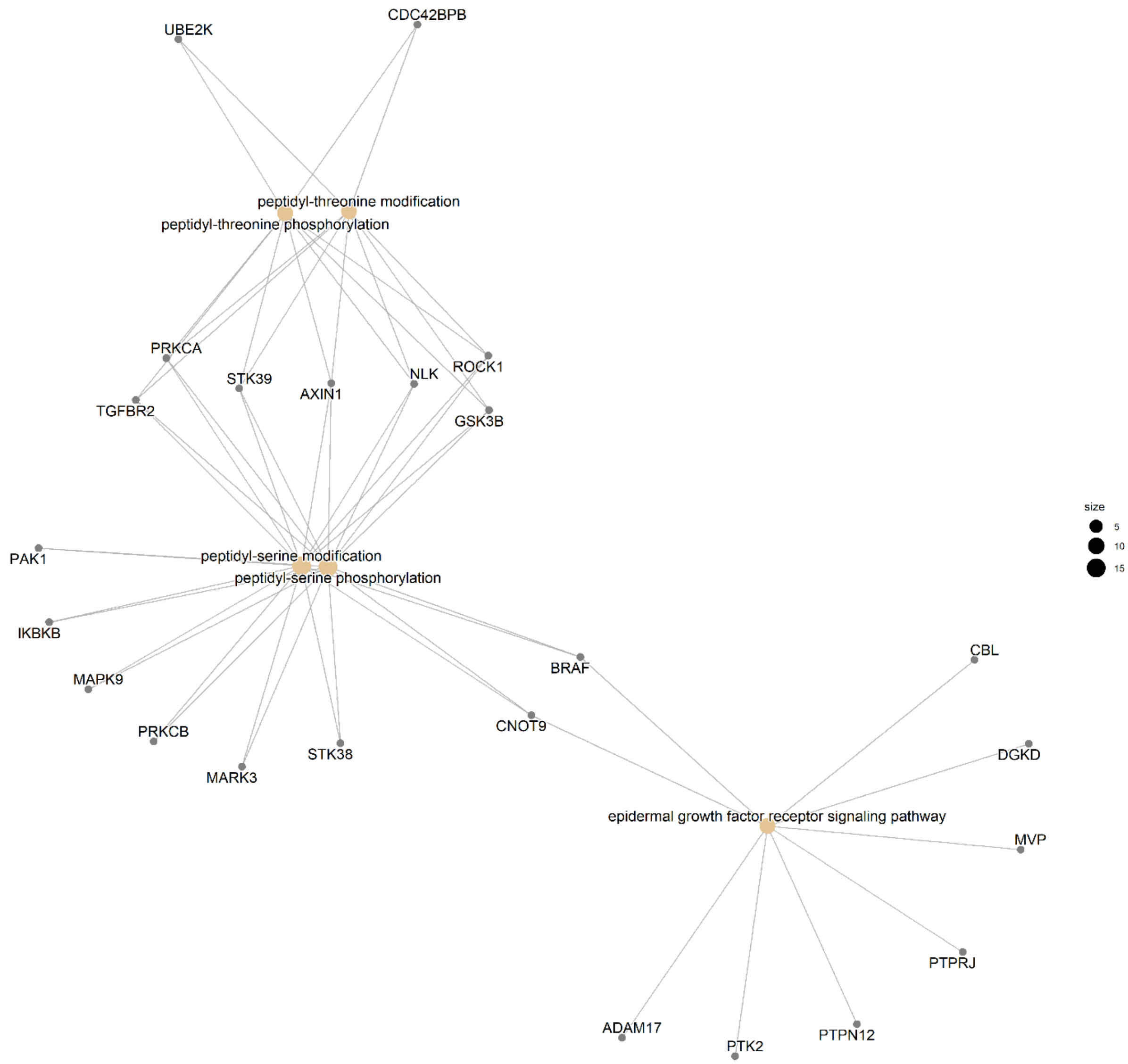

Supplementary Figure S15. Gene Ontology biological process category network plot showing relationships among enriched terms significantly associated with host genes of upregulated circRNAs in the STEMI vs Control comparison. Connected terms share contributing genes, highlighting coordinated functional modules linked to circRNA-associated changes.

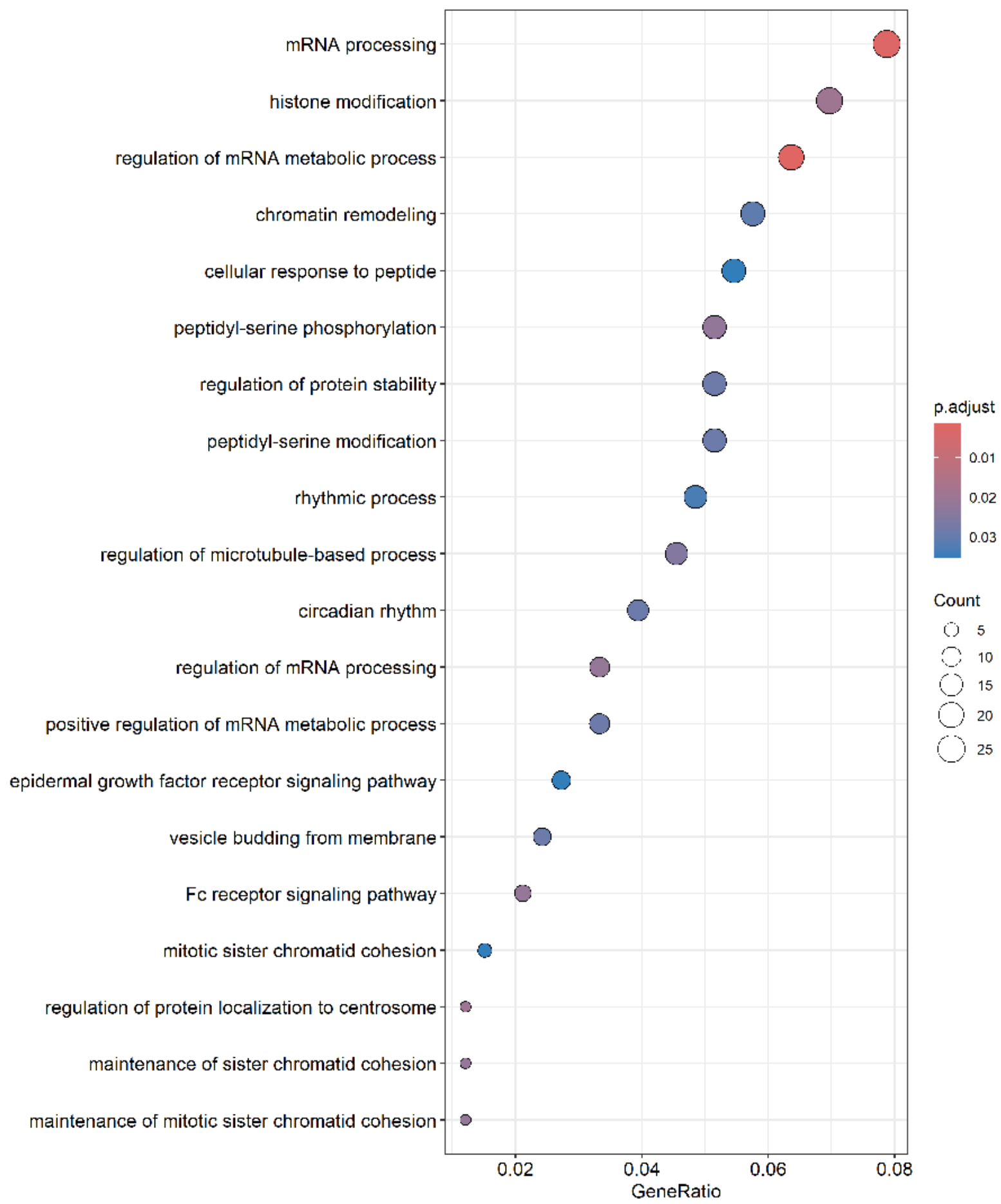

Supplementary Figure S16. Gene Ontology biological process enrichment dot plot showing terms significantly associated with host genes of upregulated circRNAs in the NSTEMI vs Control comparison. The plot summarizes enriched functional categories and provides pathway-level context for circRNA-associated changes in this contrast.

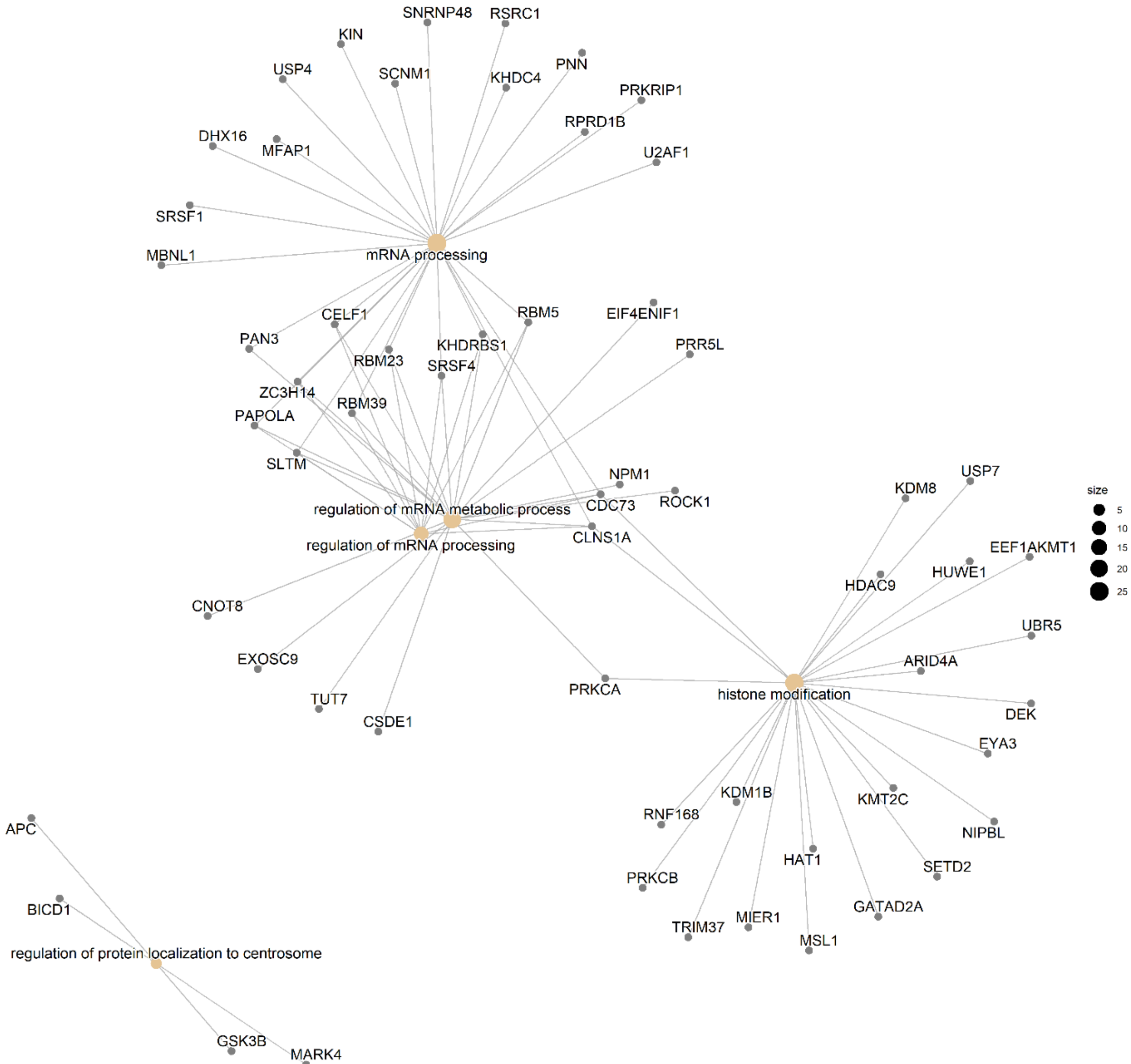

Supplementary Figure S17. Gene Ontology biological process category network plot showing relationships among enriched terms significantly associated with host genes of upregulated circRNAs in the NSTEMI vs Control comparison. Connected terms share contributing genes, highlighting coordinated functional modules linked to circRNA-associated changes.

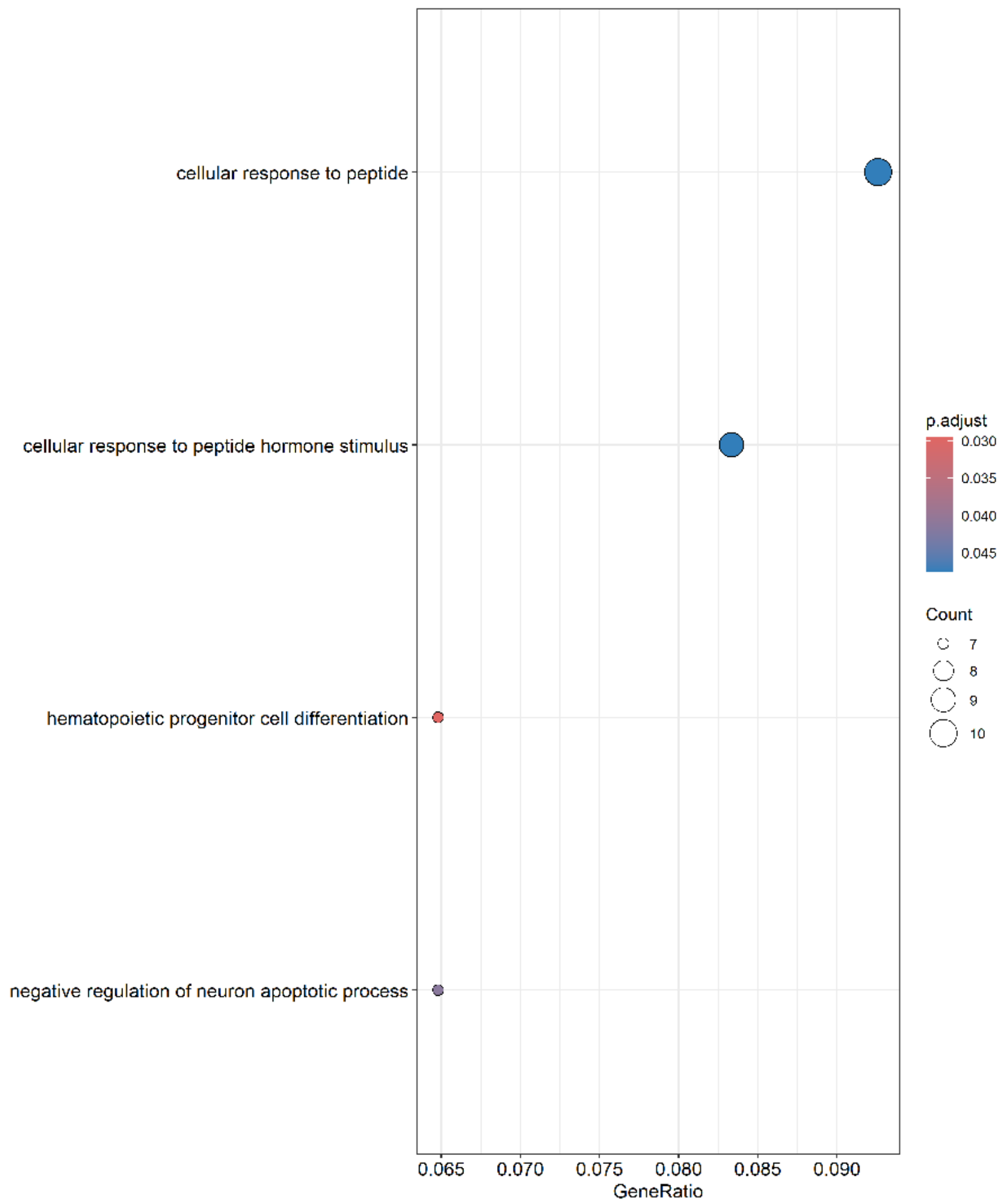

Supplementary Figure S18. Gene Ontology biological process enrichment dot plot showing terms significantly associated with host genes of upregulated circRNAs in the STEMI vs NSTEMI comparison. The plot summarizes enriched functional categories and provides pathway-level context for circRNA-associated changes in this contrast.

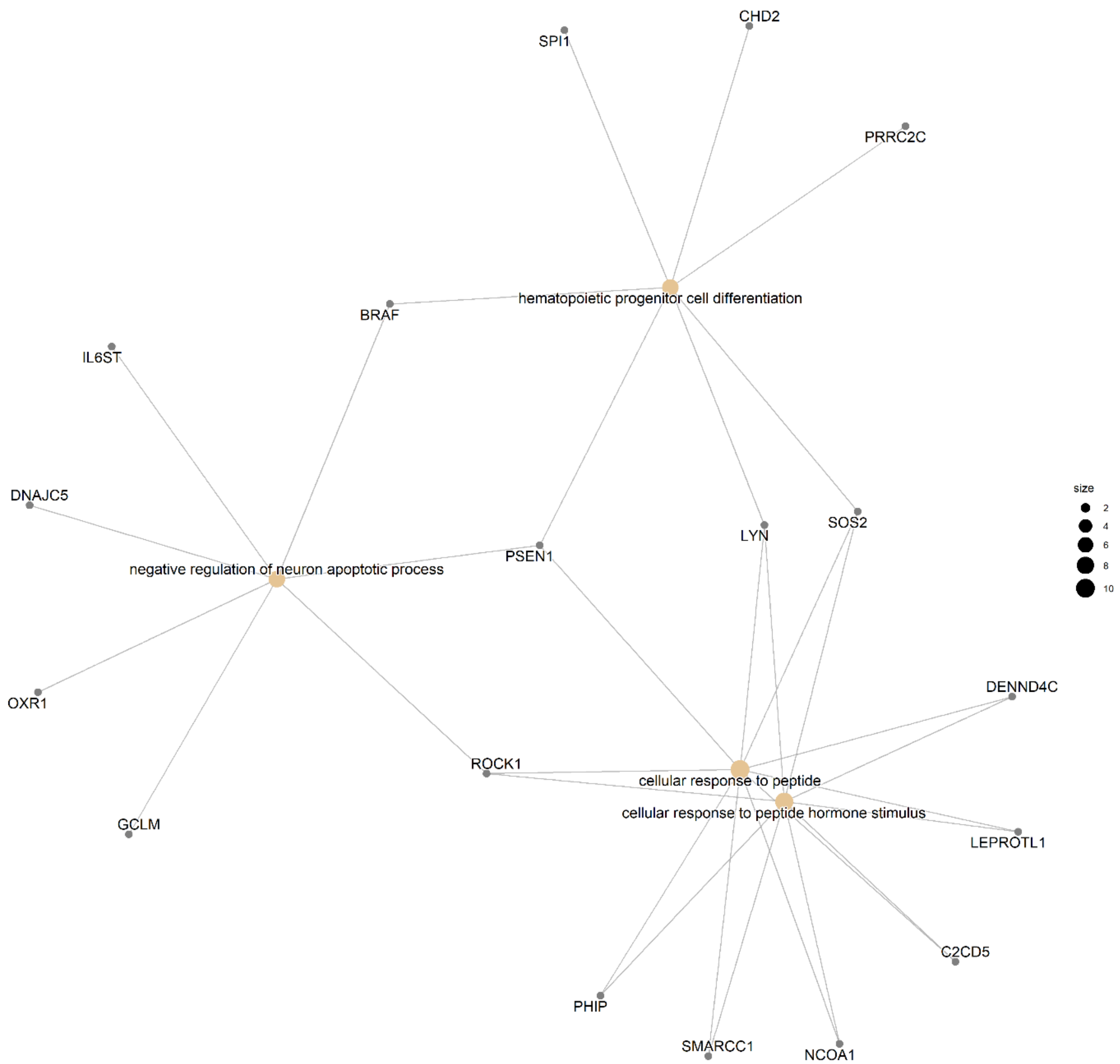

Supplementary Figure S19. Gene Ontology biological process category network plot showing relationships among enriched terms significantly associated with host genes of upregulated circRNAs in the STEMI vs NSTEMI comparison. Connected terms share contributing genes, highlighting coordinated functional modules linked to circRNA-associated changes.

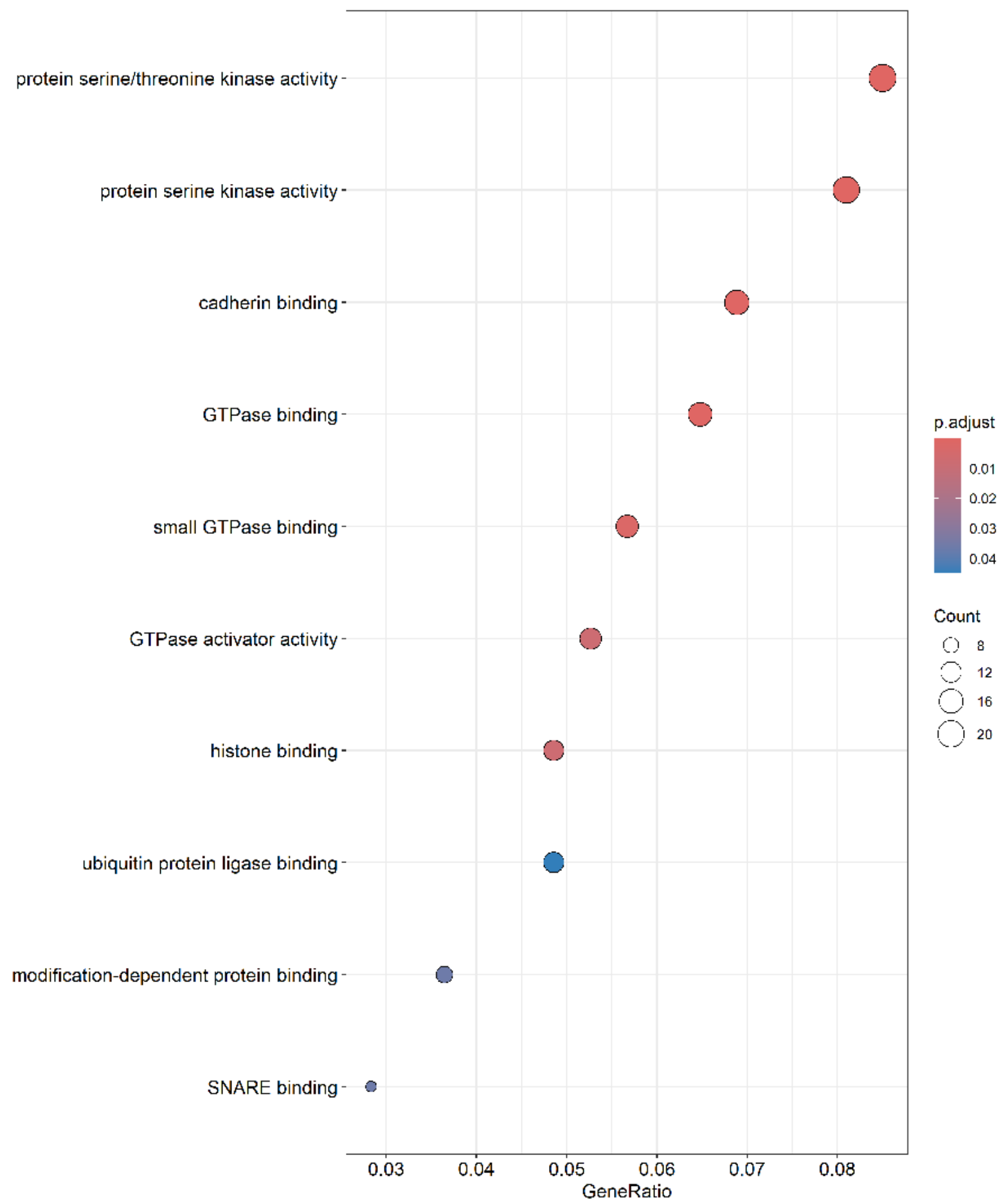

Supplementary Figure S20. Gene Ontology molecular function enrichment dot plot showing terms significantly associated with host genes of upregulated circRNAs in the STEMI vs Control comparison. The plot summarizes enriched functional categories and provides pathway-level context for circRNA-associated changes in this contrast.

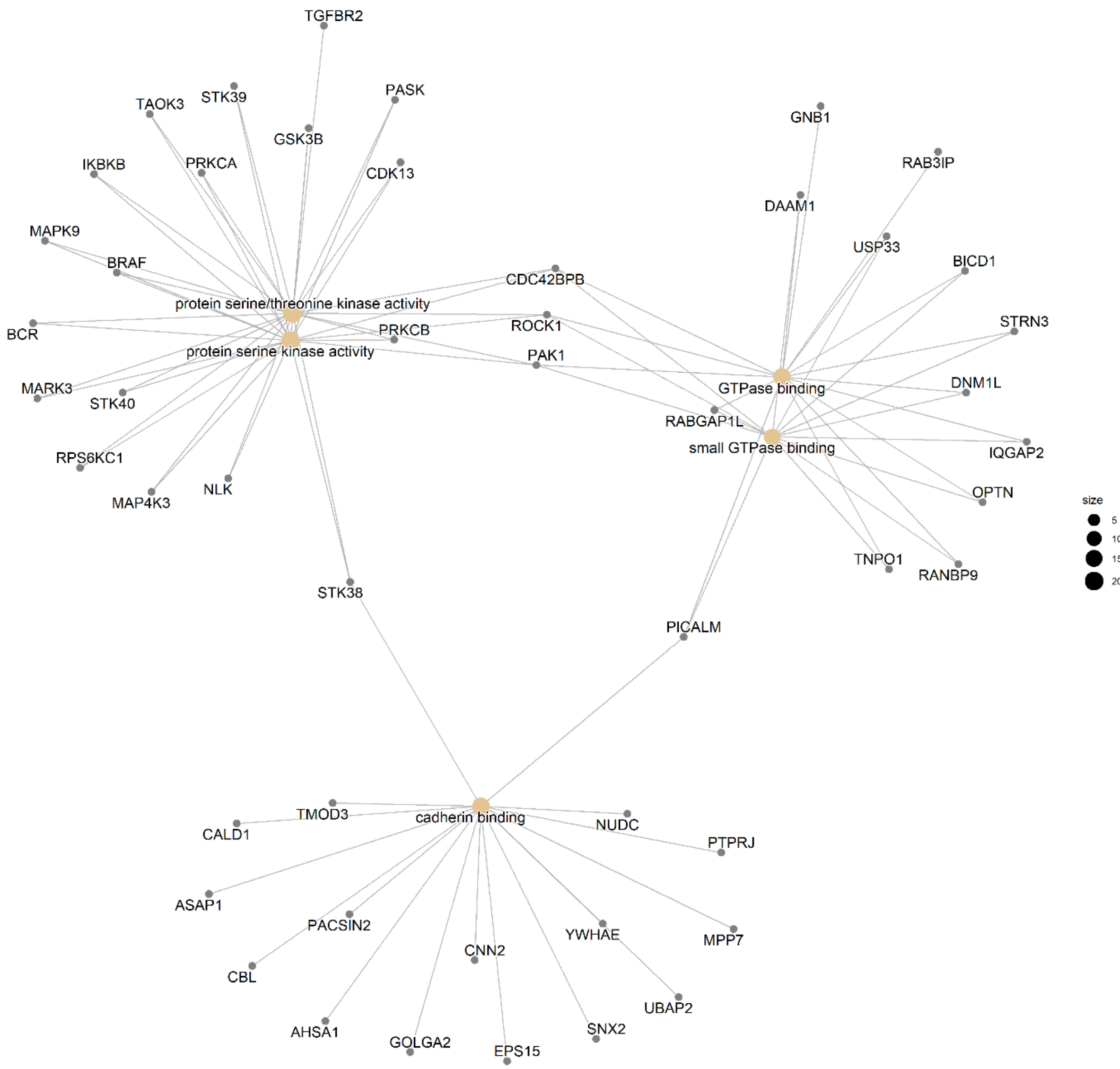

Supplementary Figure S21. Gene Ontology molecular function category network plot showing relationships among enriched terms significantly associated with host genes of upregulated circRNAs in the STEMI vs Control comparison. Connected terms share contributing genes, highlighting coordinated functional modules linked to circRNA-associated changes.

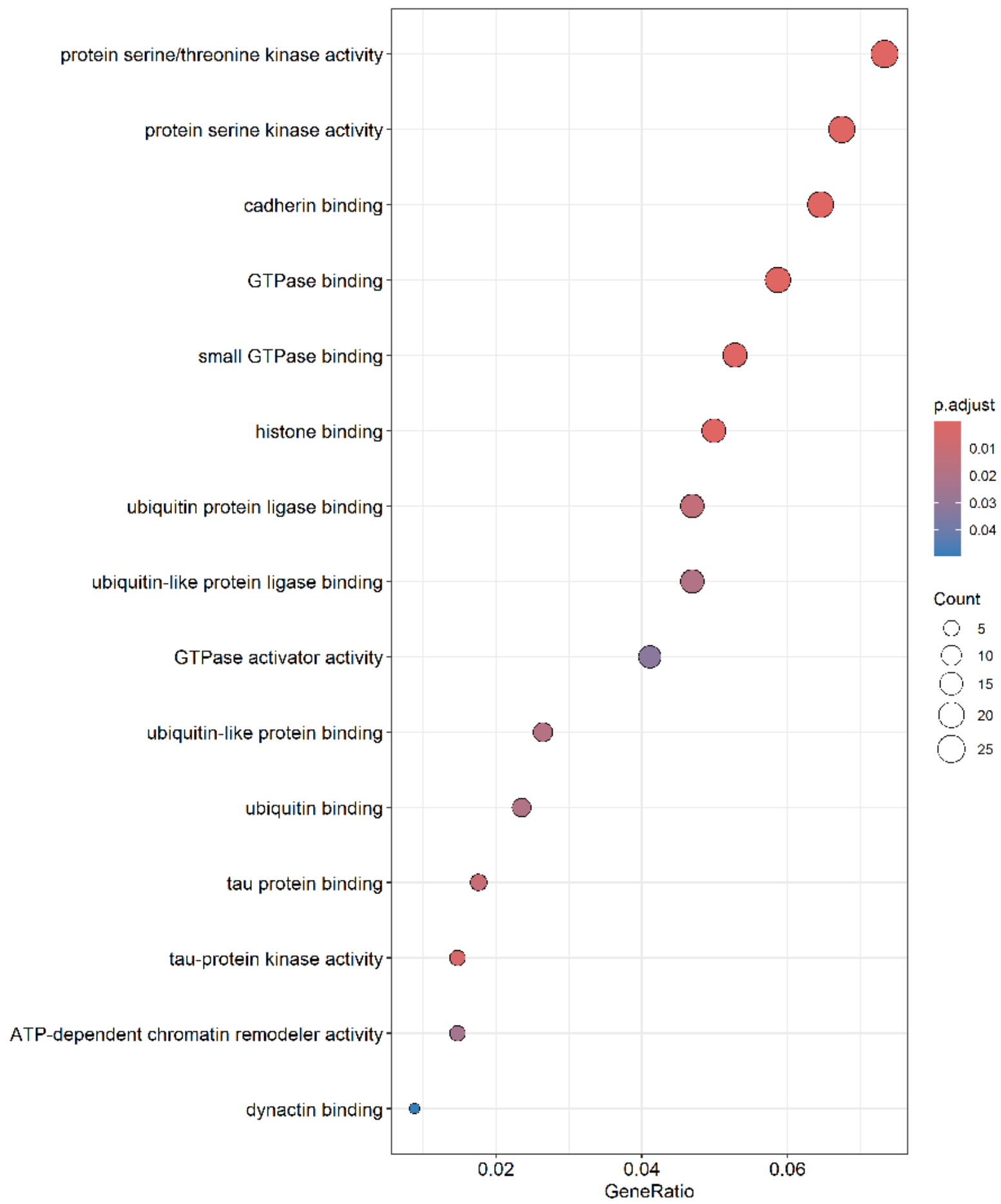

Supplementary Figure S22. Gene Ontology molecular function enrichment dot plot showing terms significantly associated with host genes of upregulated circRNAs in the NSTEMI vs Control comparison. The plot summarizes enriched functional categories and provides pathway-level context for circRNA-associated changes in this contrast.

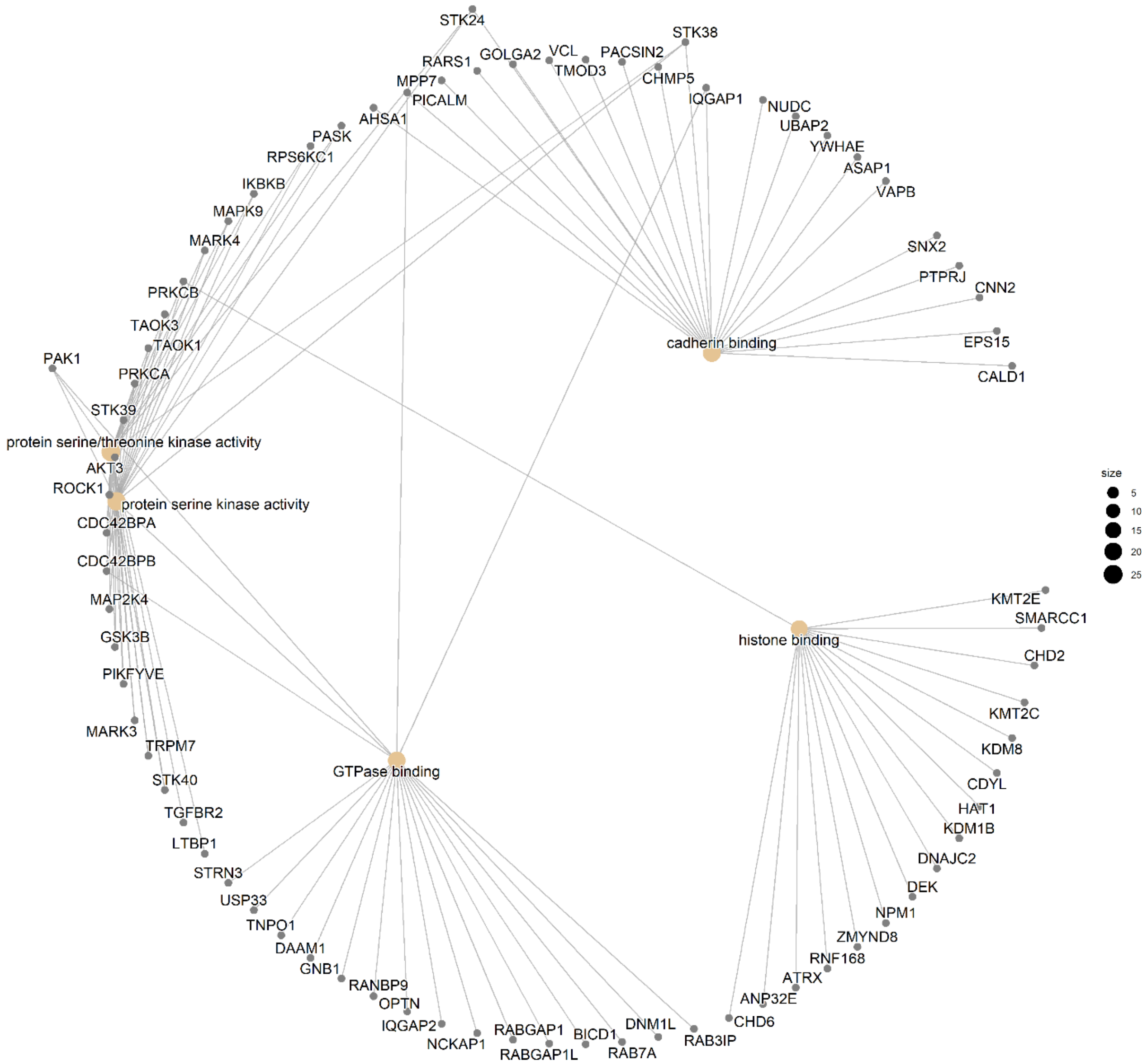

Supplementary Figure S23. Gene Ontology molecular function category network plot showing relationships among enriched terms significantly associated with host genes of upregulated circRNAs in the NSTEMI vs Control comparison. Connected terms share contributing genes, highlighting coordinated functional modules linked to circRNA-associated changes.

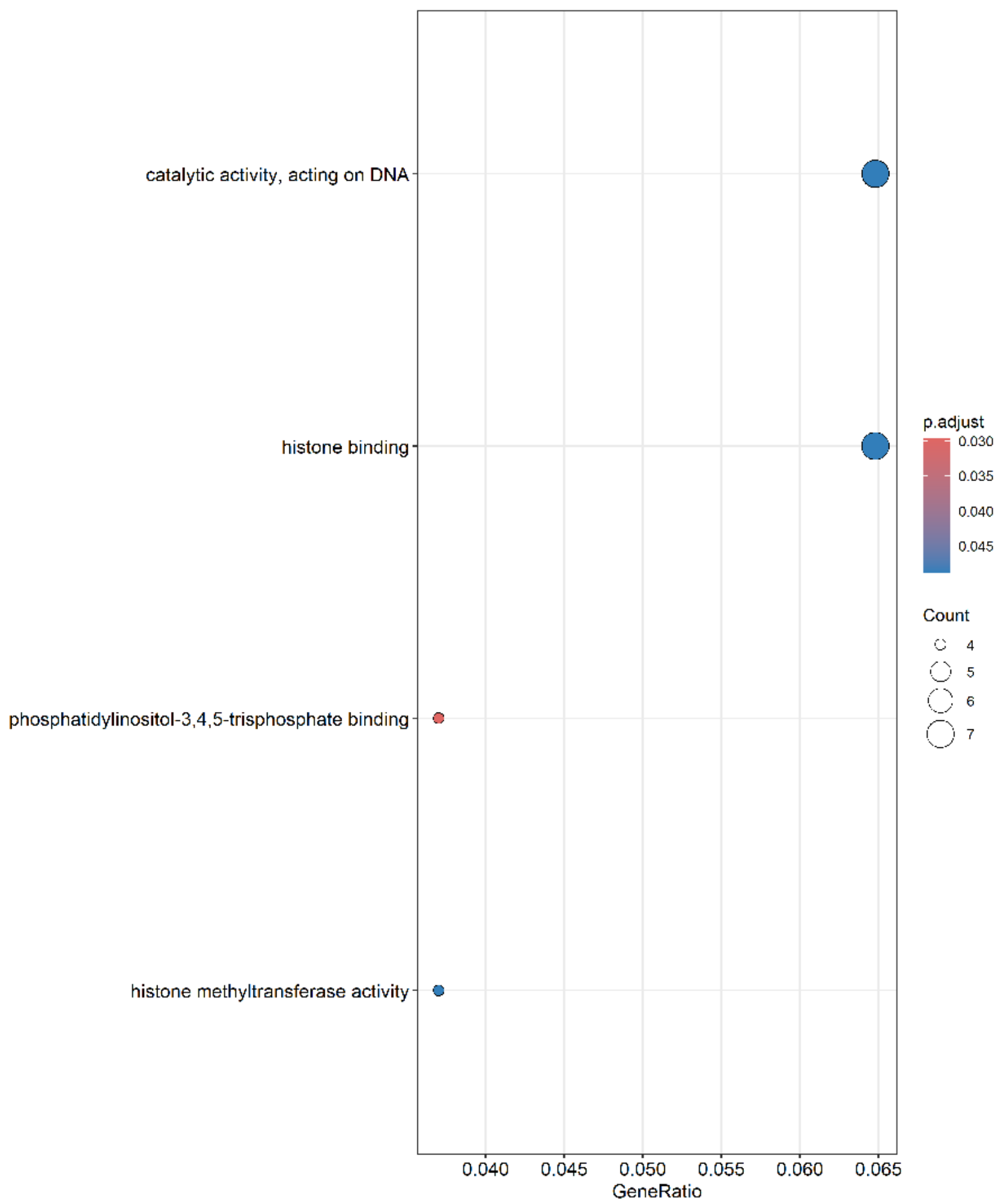

Supplementary Figure S24. Gene Ontology molecular function enrichment dot plot showing terms significantly associated with host genes of upregulated circRNAs in the STEMI vs NSTEMI comparison. The plot summarizes enriched functional categories and provides pathway-level context for circRNA-associated changes in this contrast.

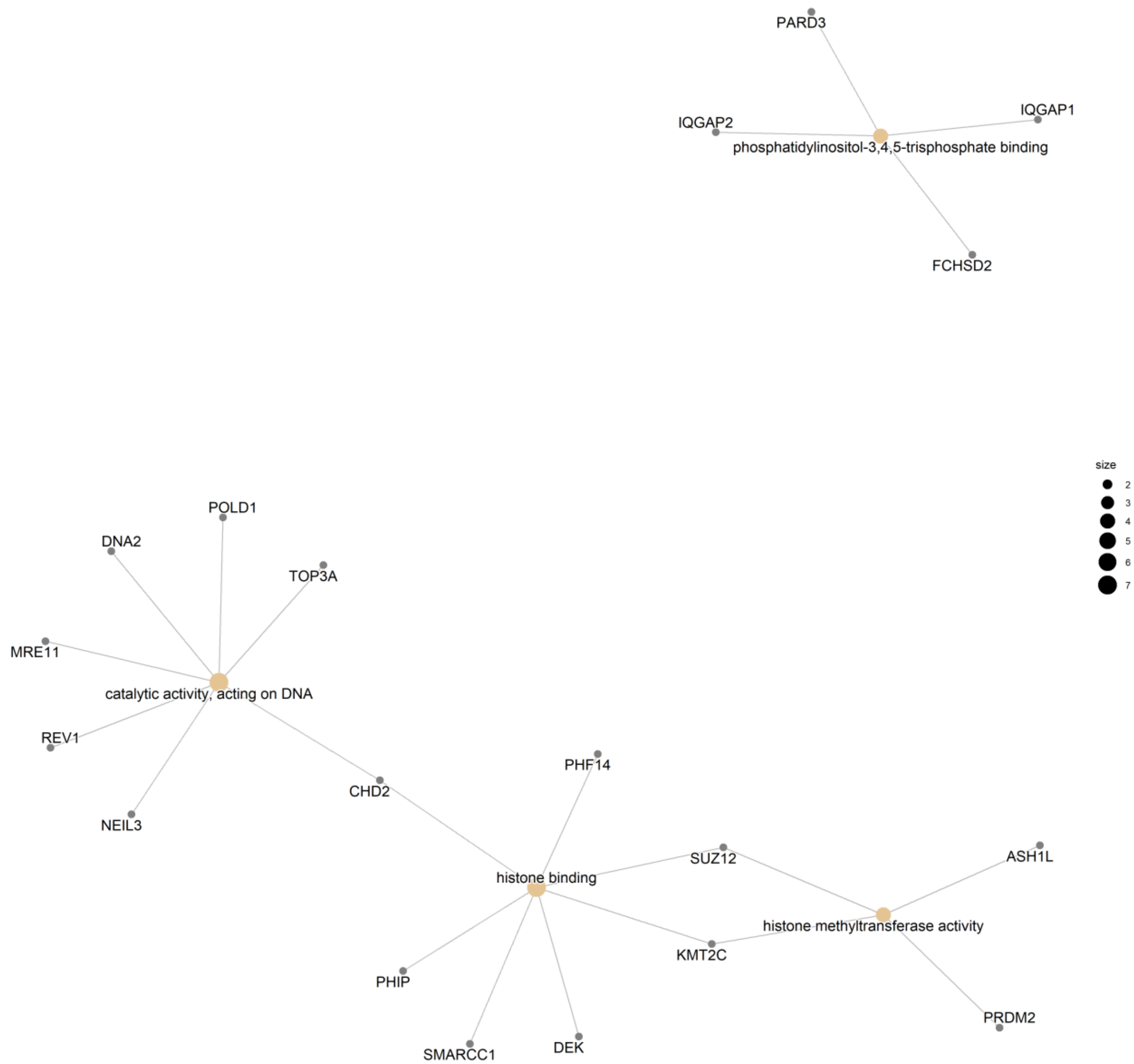

Supplementary Figure S25. Gene Ontology molecular function category network plot showing relationships among enriched terms significantly associated with host genes of upregulated circRNAs in the STEMI vs NSTEMI comparison. Connected terms share contributing genes, highlighting coordinated functional modules linked to circRNA-associated changes.

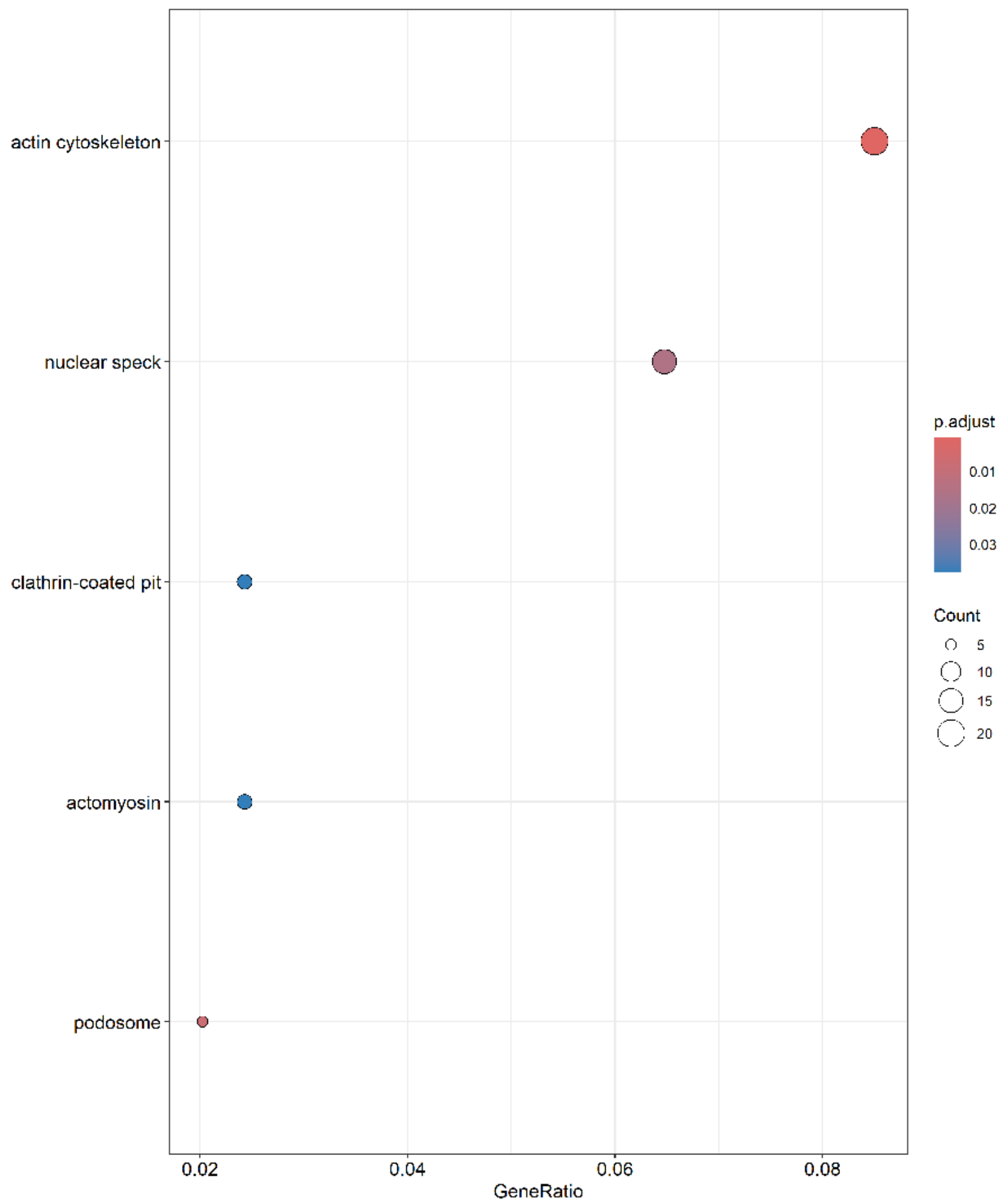

Supplementary Figure S26. Gene Ontology cellular component enrichment dot plot showing terms significantly associated with host genes of upregulated circRNAs in the STEMI vs Control comparison. The plot summarizes enriched functional categories and provides pathway-level context for circRNA-associated changes in this contrast.

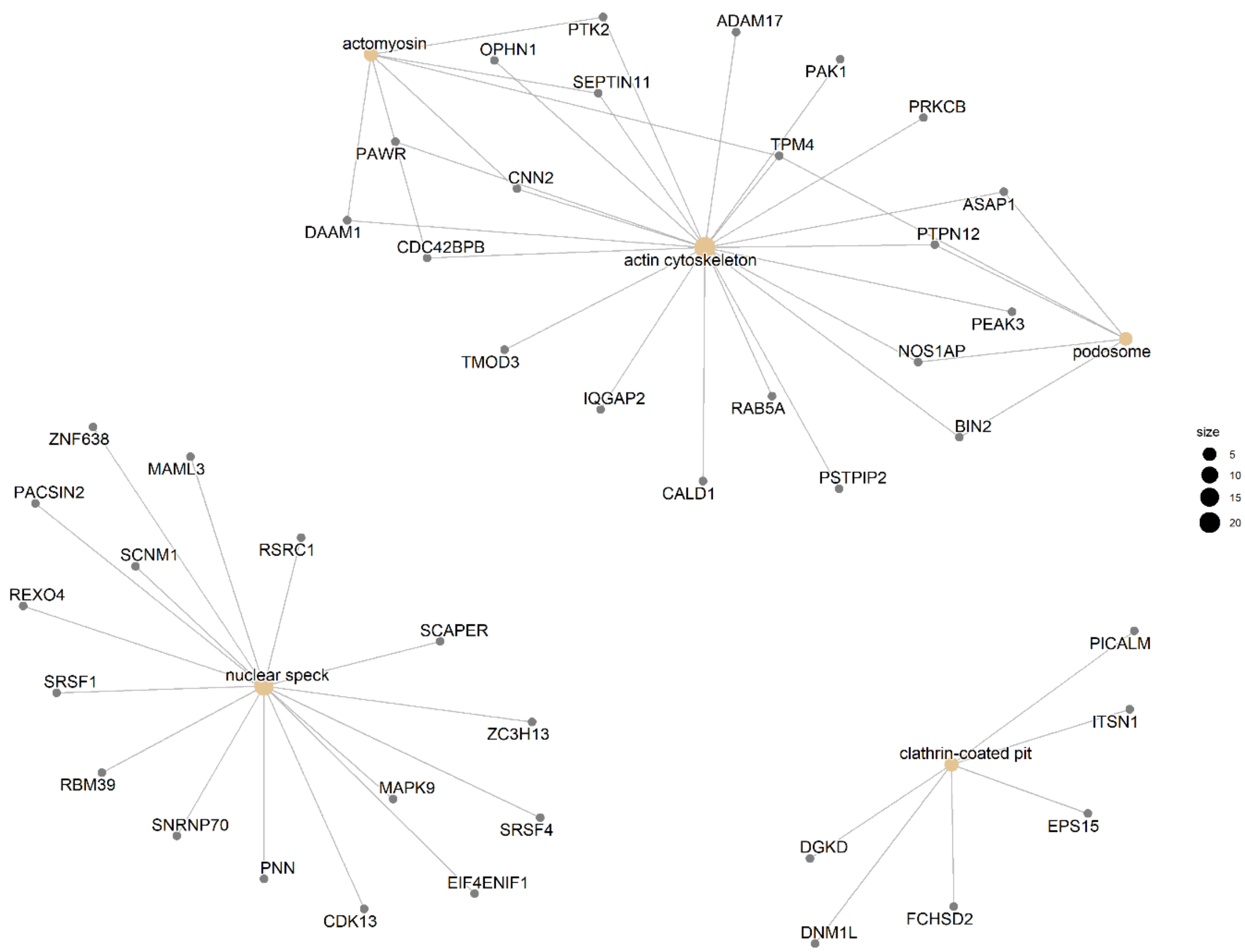

Supplementary Figure S27. Gene Ontology cellular component category network plot showing relationships among enriched terms significantly associated with host genes of upregulated circRNAs in the STEMI vs Control comparison. Connected terms share contributing genes, highlighting coordinated functional modules linked to circRNA-associated changes.

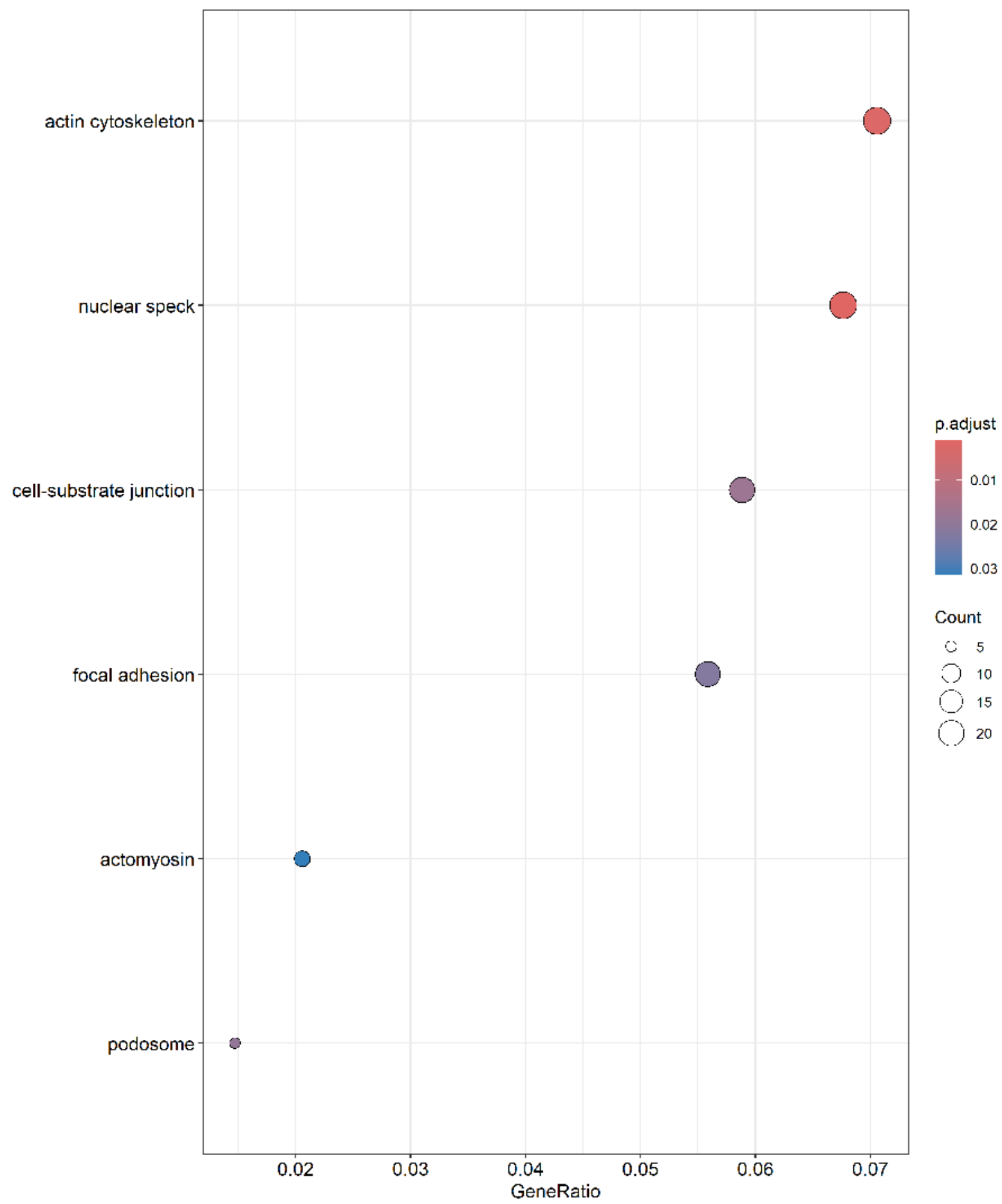

Supplementary Figure S28. Gene Ontology cellular component enrichment dot plot showing terms significantly associated with host genes of upregulated circRNAs in the NSTEMI vs Control comparison. The plot summarizes enriched functional categories and provides pathway-level context for circRNA-associated changes in this contrast.

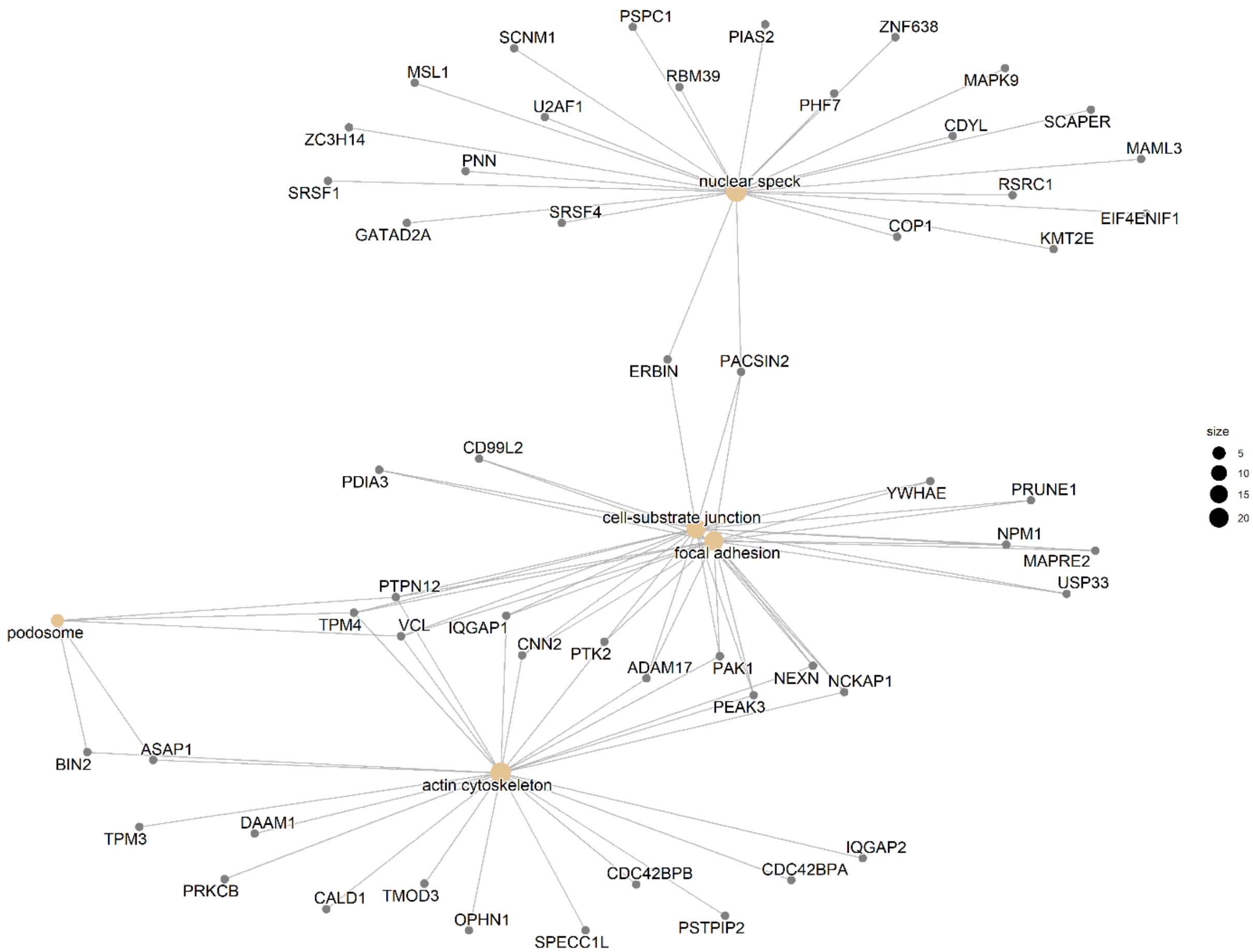

Supplementary Figure S29. Gene Ontology cellular component category network plot showing relationships among enriched terms significantly associated with host genes of upregulated circRNAs in the NSTEMI vs Control comparison. Connected terms share contributing genes, highlighting coordinated functional modules linked to circRNA-associated changes.
